# Plasma lipidomic alterations during pathogenic SIV infection with and without antiretroviral therapy

**DOI:** 10.3389/fimmu.2025.1475160

**Published:** 2025-03-10

**Authors:** Sindhuja Sivanandham, Ranjit Sivanandham, Cuiling Xu, Jen Symmonds, Paola Sette, Tianyu He, Nicholas Funderburg, Mohamed Abdel-Mohsen, Alan Landay, Cristian Apetrei, Ivona Pandrea

**Affiliations:** ^1^ Department of Pathology, School of Medicine, University of Pittsburgh, Pittsburgh, PA, United States; ^2^ Division of Infectious Diseases, Department of Medicine, School of Medicine, University of Pittsburgh, Pittsburgh, PA, United States; ^3^ Department of Infectious Diseases and Microbiology, Graduate School of Public Health, University of Pittsburgh, Pittsburgh, PA, United States; ^4^ Division of Medical Laboratory Science, School of Health and Rehabilitation Sciences, The Ohio State University, Columbus, OH, United States; ^5^ Vaccine and Immunotherapy Center, Wistar Institute, Philadelphia, PA, United States; ^6^ Department of Internal Medicine, University of Texas Medical Branch, Galveston, TX, United States

**Keywords:** simian immunodeficiency virus (SIV), human immunodeficiency virus (HIV), lipidomics, HIV comorbidities, antiretroviral therapy (ART), cardiovascular disease, metabolic disease

## Abstract

**Introduction:**

Lipid profiles change in human immunodeficiency virus (HIV) infection and correlate with inflammation. Lipidomic alterations are impacted by multiple non-HIV-related behavioral risk factors; thus, use of animal models in which these behavioral factors are controlled may inform on the specific lipid changes induced by simian immunodeficiency virus (SIV) infection and/or antiretroviral therapy (ART).

**Methods:**

Using ultrahigh Performance Liquid Chromatography-Tandem Mass Spectroscopy, we assessed and compared (ANOVA) longitudinal lipid changes in naïve and ART-treated SIV-infected pigtailed macaques (PTMs). Key parameters of infection (IL-6, TNFa, D-dimer, CRP and CD4^+^ T cell counts) were correlated (Spearman) with lipid concentrations at critical time points of infection and treatment.

**Results:**

Sphingomyelins (SM) and lactosylceramides (LCER) increased during acute infection, returning to baseline during chronic infection; Hexosylceramides (HCER) increased throughout infection, being normalized with prolonged ART; Phosphatidylinositols (PI) and lysophosphatidylcholines (LPC) decreased with SIV infection and did not return to normal with ART; Phosphatidylethanolamines (PE), lysophosphatidylethanolamines (LPE) and phosphatidylcholines (PC) were unchanged by SIV infection, yet significantly decreased throughout ART. Specific lipid species (SLS) were also substantially modified by SIV and/or ART in most lipid classes. In conclusion, using a metabolically controlled model, we identified specific lipidomics signatures of SIV infection and/or ART, some of which were similar to people living with HIV (PWH). Many SLS were identical to those involved in development of organ dysfunctions encountered in virally suppressed individuals. Lipid changes also correlated with markers of disease progression, inflammation and coagulation.

**Discussion:**

Our data suggest that lipidomic profile alterations contribute to residual systemic inflammation and comorbidities seen in HIV/SIV infections and therefore may be used as biomarkers of SIV/HIV comorbidities. Further exploration into the benefits of interventions targeting dyslipidemia is needed for the prevention HIV-related comorbidities.

## 1 Introduction

Over the last few decades, tremendous advances in antiretroviral therapy (ART) and improved policies for the management of HIV infection have resulted in a spectacular increase in the life expectancy of people living with HIV (PWH) (1, 2). Altogether, these advances transformed HIV infection from an implacably deteriorating severe disease with a high mortality rate, into a chronic disease with a nearly normal life span (3). Despite these increases in life expectancy, the overall mortality of PWH is still three times higher than that of the general population (4). Additionally, as PWH age, more are experiencing non-AIDS-related comorbidities, that significantly impact their lifespan and quality of life (5–8). Cardiovascular (CVD), hepatic, renal, pulmonary diseases, as well as cognitive disorders also occur more frequently in younger PWH (5, 9–11), indicating an accelerated aging in this population (11, 12).

HIV-associated comorbidities can be partly due to a plethora of risk factors, such as demographics (race, ethnicity, and socioeconomic status) (5), HIV itself, specific classes of antiretrovirals (ARVs) or behavioral factors (13). However, the exact mechanisms of HIV-associated comorbidities and the extent to which non-AIDS-defining illnesses are independent comorbidities, are due to HIV infection, behavioral factors, or ART itself has yet to be determined (5).This is difficult to accomplish due to numerous confounder factors that may impact clinical studies involving PWH cohorts.

The multifactorial nature of behavioral determinants of HIV comorbidities is illustrated by the higher frequency of metabolic disturbances, such as insulin resistance, hyperinsulinemia, hypercholesterolemia, hypertriglyceridemia, low levels of high-density lipoprotein cholesterol (HDL-C) and truncal adiposity (HIV-associated lipodystrophy) in PWH than in individuals without HIV of similar age and weight (13, 14). Dyslipidemia reported for PWH may be caused by both HIV (15), and ART (15, 16), which could both also independently contribute to decreased insulin sensitivity (15). As such, the increased risk for CVD in PWH (8, 17) can be attributed not only to chronic inflammation and immune dysfunction (18), but also to metabolic disturbances induced by the ART itself (13, 16).

The field of lipidomics is rapidly emerging and has a wide range of impending applications, potentially identifying new pathogenic pathways, biomarkers for various clinical conditions, and putative therapeutic targets (19).

Lipids are components of cell membranes, and have key structural and barrier roles (19, 20), being also involved in cell proliferation and survival (21, 22), cellular signaling mechanisms (21), energy metabolism (21), calcium homeostasis (21), membrane trafficking (23, 24), cytoskeletal organization (23), apoptosis (25, 26), aging (27), autophagy (28), migration of immune cells (29) and several other metabolic pathways, all of which are vital for physiological homeostasis. The biological properties of lipids depend on their chemical structure, and as individual lipid molecules have unique physicochemical properties, each lipid species has distinctive functions (30).

The advent of high resolution, high sensitivity and high mass accuracy mass spectrometers enabled us to identify hundreds of lipid species in a single sample (19). Lipids from multiple tissues and cells can be identified and quantified (19), and since body fluids also yield a remarkable lipid diversity (19), plasma lipidomic profiling of specific disease-induced changes allows for noninvasive diagnosis of diseases and comorbidities with minimal patient discomfort.

Lipidome imbalances are associated with multiple disease (30): atherosclerosis and CVD (31, 32), diabetes (21), neurological disorders (21), infectious diseases (33, 34), renal disorders (35), hepatic disorders (36), and cancers (21). In all these diseases, lipidome assessment can provide insight into the metabolic changes that may underly the causative disease processes. HIV infection is no exception: the multiple lipid functions and their involvement in multiple pathogenic pathways (19) point to the HIV-associated lipidomic changes as potential triggers and key biomarkers of different HIV-associated comorbidities. Yet, few studies reported changes in several lipid classes in PWH and suggested that they are associated with both residual inflammation and development of comorbidities in these patients (37–39).

Lipid metabolism can however change with everyday activities, such as diet, exercise, environment and also by various other behavioral and clinical conditions (19, 40–42). Therefore, use of models with minimal confounding variables is necessary to assess fine changes that would otherwise be missed. Nonhuman primates (NHPs) are ideal for the study of HIV and ART-related comorbidities (8, 43), as they enable studies in a controlled environment, with the same diet and physical activity, while being completely free of tobacco, alcohol and recreational drugs, thereby minimizing the impact of confounding factors in this system. The NHP model also gives us the ability to compare the uninfected, untreated and treated states in the same individual thus allowing us to focus on uncovering the real impact of SIV infection and ART on the lipidome status (8). Finally, the NHPs have a major advantage compared with other models (i.e., rodents) for the lipidomic studies, as monkey genetics is closer to that of humans (44–46).

We assessed the changes in plasma lipid species triggered by SIV infection and attempted to differentiate these changes as being consequential to the retroviral infection itself or to the pharmacodynamics of ART. We also report reversibility of some SIV-related lipidome changes in NHPs on ART. Identifying and understanding lipidome alterations and pathways of reversibility characteristic to SIV infection and ART, and distinguishing their determinants can provide insight into and validate the factors driving comorbidities in PWH. This may also allow identification of prognostic markers for early detection and prevention of comorbidities in PWH and new therapeutic avenues to tackle HIV-related comorbidities. Such studies may lay the ground to not only a near-normal life span for PWH, but also to a healthier, morbidity-free course of HIV infection.

## 2 Results

### 2.1 Animal groups and study design

**Figure 1:**
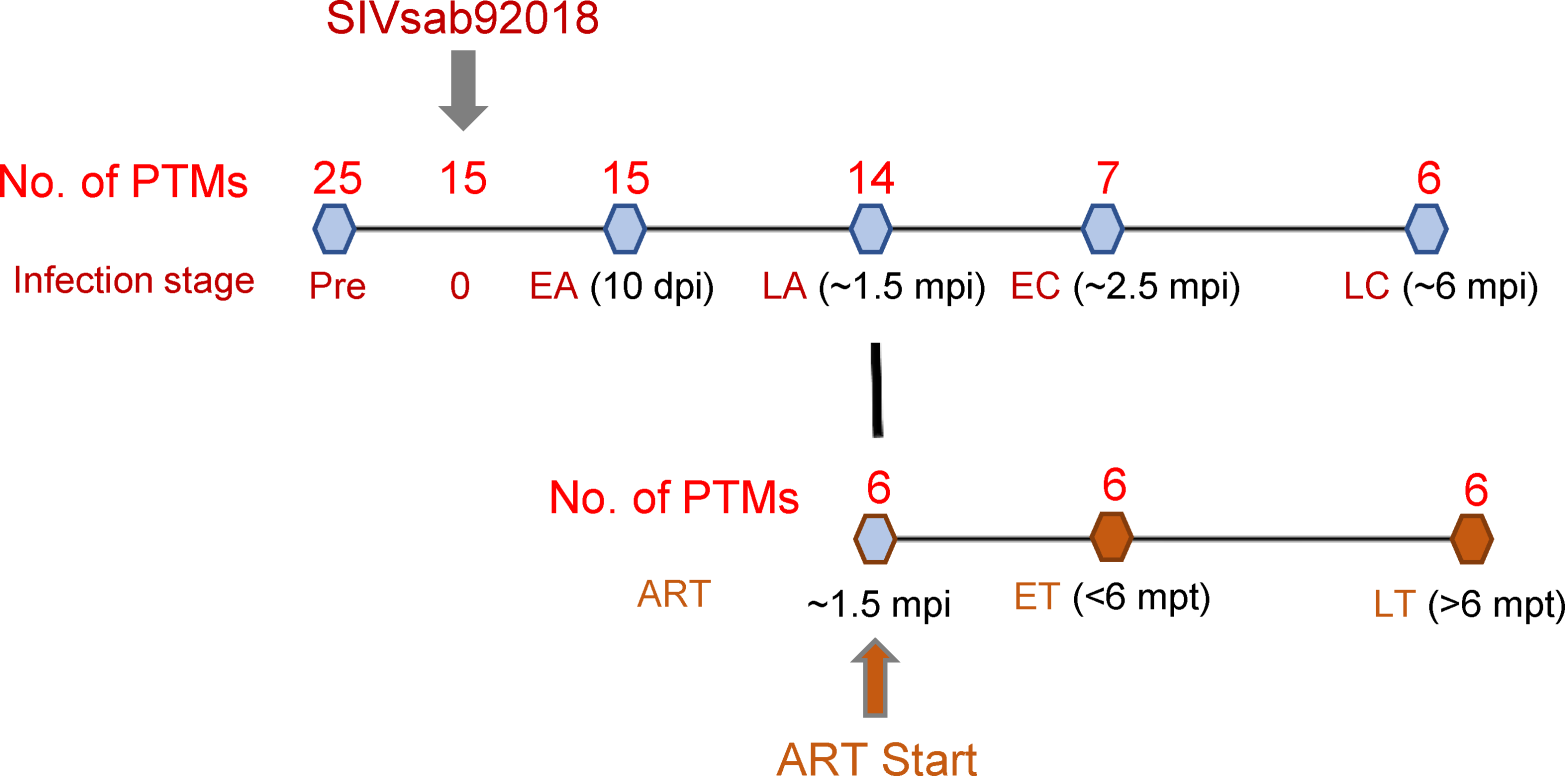
Study design. Twenty-five pigtailed macaques (PTMs) were included, 18 were infected with SIVsab92018, and six were given antiretroviral therapy (ART) from ~ 1.5 months postinfection (mpi) onward. They were sampled at the following time points: preinfection (all 25 PTMs), 10 days postinfection (dpi) (n=15); at ~ 1.5 mpi, a time point corresponding to late acute infection (LA) (n=14); at ~ 2.5 mpi, a time point corresponding to early chronic infection (EC) (n=7); at ~ 6 mpi, a time point corresponding to late chronic infection (LC) (n=6). The lipidomic changes induced by ART were assessed in the 6 PTMs on ART during early ART, i.e., < 6 months post treatment (mpt; ET) and during late treatment, i.e., >6 mpt (LT).

Samples from twenty-five pigtailed macaques (*Macaca nemestrina*; PTMs) were included. Fifteen of the twenty-five were intravenously infected with 300 TCID50 of SIVsab (47). Starting from ~1.5 months postinfection (mpi), six received coformulated ART (48) throughout the remainder of the follow-up. Coformulated ART consisted of a combination of first line antiretrovirals used in PWH (Tenofovir+Emtricitabine+Dolutegravir). The following blood samples were available for lipidome analyses: preinfection samples for twenty-five uninfected PTMs; infection time point (D0) and early acute (EA) infection [~10 days postinfection (dpi)] for fifteen PTMs; late acute (LA) infection (~1.5 mpi) for fourteen PTMs; early chronic (EC) infection [~2.5 mpi] for seven PTMs; and late chronic (LC) infection (~6 mpi) for six PTMs. Assessment of the impact of SIV infection on the lipidome profile was performed by comparing each timepoint to the overall preinfection baseline levels (**Figure 1**; **Supplementary Table 1**).

The assessment of the overall effect of SIV and ART on lipidome was performed by comparing the lipidomics profiles in the blood samples collected at <6 mpt (~2.5 mpt or >6 mpt (~10 mpt) to the pretreatment baselines from six SIV-infected PTMs on ART. In the text, we refer to <6 months as early treatment (ET) and >6 months as late treatment (LT) timepoints (**Figure 1**; **Supplementary Table 1**).

### 2.2 Lipidome assessment

Similar to humans, the lipidomic analysis identified 14 lipid classes. We first screened for significant variations in concentration of the various lipid classes and then of species in each class throughout infection, and with ART. The lipid species that yielded altered concentrations during SIV infection or ART were next correlated with key biomarkers of SIV/HIV disease progression and comorbidities.

### 2.3 Total lipidome analyses

**Figure 2:**
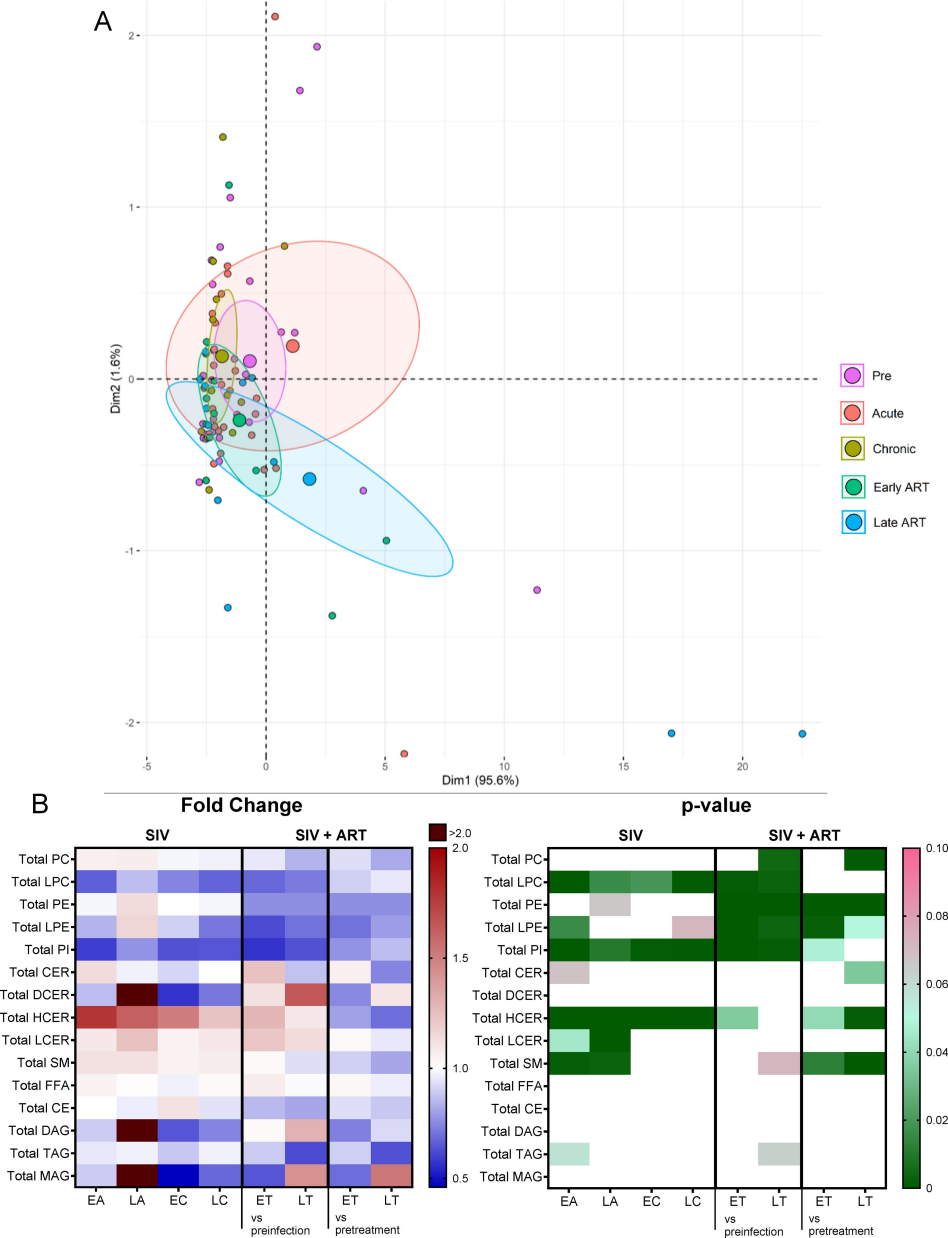
Principal component analysis (PCA) plot showing overall lipidome variation based on concentrations of totals of lipid classes, with confidence ellipses plotted for each group **(A)**. Groups are defined as preinfection, early acute infection (EA); late acute infection (LA), early chronic infection (EC), late chronic infection (LC), early ART (ET), late ART (LT). **(B)** Heatmaps of fold changes and p-values of totals of lipid classes showing changes with SIV infection, where EA, LA, EC and LC are compared with preinfection, and changes with ART, where ET and LT are compared with preinfection, and then with pretreatment. For fold change, red represents fold increase with deeper intensity indicating greater increase, and blue represents fold decrease with deeper intensity indicating greater decrease. White indicates no fold change. For p-value, green indicates statistically significant change (p<0.05) with deeper color intensity indicating stronger significance, and pink indicates trend to significance (p<0.1) with deeper color intensity indicating weaker significance.

Changes in the overall lipidome were observed at every stage of SIV infection, and with ART (**Figure 2A**), with the key lipid classes being modified as follows (**Figure 2B**): (i) The levels of sphingomyelins (SM) and lactosylceramides (LCER) increased only during acute SIV infection, returning to baseline preinfection during chronic infection; (ii) Hexosylceramides (HCER) increased throughout SIV infection and returned to baseline with prolonged ART; (iii) phosphatidylinositols (PI) and lysophosphatidylcholines (LPC) decreased with SIV infection and ART did not normalize their overall levels during the follow-up; (iv) Phosphatidylethanolamines (PE), lysophosphatidylethanolamines (LPE) and phosphatidylcholines (PC) did not change with SIV infection, yet they were reduced throughout ART.

Conversely, dihydroceramides (DCER), free fatty acids (FFA), cholesterylesters (CE), triacylglycerols (TAG), diacylglycerols (DAG) and monoacylglycerols (MAG) remained unaltered with SIV and ART (**Figure 2B**).

We therefore further focused the analyses just on the lipid species belonging to lipid classes that showed significant variation with SIV and/or ART.

#### 2.3.1 Phosphatidylcholines

*Phosphatidylcholines (PCs)* comprise half of the total cellular phospholipids of mammalian cells and subcellular organelles (49), being essential to lipoprotein formation and stabilization. Three-fourths of the phospholipids on the surface of apoB lipoproteins are PCs (49). Hepatic PC biosynthesis stimulates the secretion of VLDL particles (49). Finally, PCs have anti-inflammatory effects (50).

**Figure 3:**
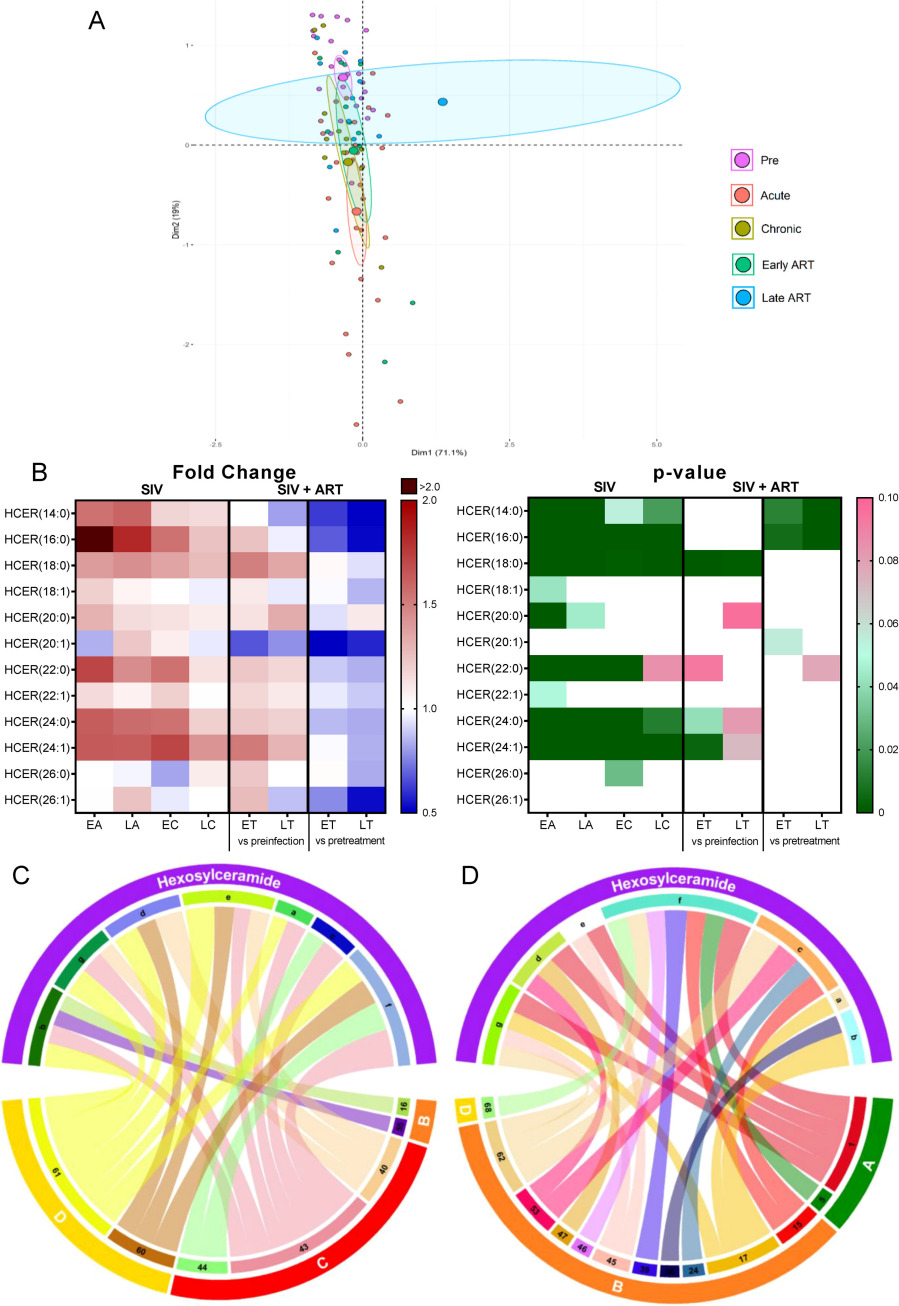
Principal component analysis (PCA) plot showing overall variation of phosphatidylcholines (PC) based on concentrations of PC species with confidence ellipses plotted for each group **(A)**. Groups are defined as preinfection, early acute infection (EA); late acute infection (LA), early chronic infection (EC), late chronic infection (LC), early ART (ET), late ART (LT). Heatmaps of fold changes and p-values of PC species changes with SIV infection, where EA, LA, EC and LC are compared with preinfection, and changes with ART, where ET and LT are compared with preinfection, and then with pretreatment **(B)**. For fold change, red represents fold increase with deeper intensity indicating greater increase, and blue represents fold decrease with deeper intensity indicating greater decrease. White indicates no fold change. For p-value, green indicates statistically significant change (p<0.05) with deeper color intensity indicating stronger significance, and pink indicates trend to significance (p<0.1) with deeper color intensity indicating weaker significance. Positive **(C)** and negative **(D)** correlations between PC species that are altered during SIV infection or with ART, represented in upper arc: a: 12:0/18:2; b: 16:0/20:3; c: 16:0/20:4; d: 16:0/22:5; e: 17:0/18:2; f: 18:0/18:1; g: 18:1/18:2; h: 18:2/18:2; i: 18:2/18:3; j: 18:2/20:2; k: 18:2/20:3; l:18:2/20:4; m: 18:2/20:5; n: 18:2/22:6; o: 20:0/18:2; p: Total PC species. PC species are correlated with blood biomarkers of SIV infection and comorbidities coded as: 2. CD4^+^ T cells (%); 4. D-Dimer; 6. Platelets/μL; 8. Lymphocytes/μL; 10. Fibroblast growth factor (FGF); 11: IL-1B; 12. Granulocyte colony-stimulating factor (G-CSF); 15. Rantes; 16. IL-8; 17. IL-4; 18. CXCL9 [Monokine induced by gamma interferon (MIG)]; 19. CXCL10 (IP-10); 20. IL-2; 21. TNF-A; 22. IL-1RA; 23. Macrophage migration inhibitory factor (MIF); 24. I-TAC; 26. INF-γ; 27. Vascular endothelial growth factor (VEGF); 28. hepatocyte growth factor (HGF); 29. IL-5; 30. epidermal growth factor (EGF); 31. IL-15; 32. CCL-2 (monocyte chemoattractant protein-1, MCP-1); 33. CCL4 [macrophage inflammatory protein 1β (MIP-1β)]; 34. Granulocyte-macrophage colony-stimulating factor (GM-CSF); 35. CCL3 [macrophage inflammatory protein 1α (MIP-1α)]; 36. IL-17; 37. CCL11 (Eotaxin); 38. IL-6, 39. Soluble tissue factor (sTF); 40. p-selectin, 42. Soluble CD14 (sCD14); 46. Neopterin; 47. sCD163; 50. CD69^+^ CD4^+^ T cells (%); 51. Ki-67^+^ CD4^+^ T cells (%); 52. CD38^+^ HLA-DR^+^ CD4^+^ T cells (%); 53. CD25^+^ CD8^+^ T cells (%); 54. CD69^+^ CD8^+^ T cells (%); 55. Ki-67^+^ CD8^+^ T cells (%); 57. Cholesterol; 58. Triglycerides; 60. Apolipoprotein A1 (apoA1); 61. Adiponectin; 67: Leptin. The biomarkers of SIV disease progression and comorbidities are represented on lower arc, and are grouped as: A: cell counts; B: T-cell immune activation/inflammation markers; C: coagulation markers; and D: atherogenic markers. Chords are plotted as a function of log of inverse of p-value (Anova). Greater the thickness of the chord, stronger the correlation.

On principal component analysis of PC species, PC levels clustered close to the baseline during acute infection yet shifted from the preinfection during chronic infection and late ART (**Figure 3A**).

The total PC levels did not change significantly from baseline levels upon SIV infection, yet they decreased with prolonged ART (**Figure 2B**).

Changes of the PC species were heterogeneous, (**Figure 3B**) thus explaining the lack of statistical significance for the total PC levels in many of the analyzed time points.

Twelve out of 111 PC species decreased throughout SIV infection (**Figure 3B**), of which 6 remained decreased on ART. The remaining 6 were restored to baseline levels on ART. Only three PC species increased with SIV infection, and two of these were normalized by ART. This emphasizes that ART restores the levels of only some PC species that are modified by SIV, while having no effect on the other species. However, eleven PC species were specifically decreased just by ART demonstrating a clear impact of the treatment on this lipid class.

CD4^+^ T cell counts, lymphocyte counts, platelet counts, and triglycerides levels positively correlated with the maximum number of PCs which decreased in SIV or ART [Ex., PC(18:2/18:3), PC(18:0/18:1)] (**Figure 3C**).

Many other PCs decreased by SIV or ART [Ex PC(18:2/20:4), PC(18:0/18:1)] negatively correlated with the greatest number of inflammatory/immune activation and coagulation markers (**Figure 3D**), suggestive of anti-inflammatory roles of these PC species. Decrease in these anti-inflammatory PC species in SIV may be thus one of the contributive factors of increased inflammation seen in SIV.

#### 2.3.2 Lysophosphatidylcholines

*Lysophosphatidylcholines (LPCs)* are membrane-derived bioactive lysophospholipids (51) with important antinflammatory and immune functions, which may act as immunoregulatory ligands for the innate and adaptive immune cells (51). However, findings from recent clinical lipidomics studies have been controversial and somewhat confusing. For example, plasma LPCs showed an inverse relationship with cardiovascular diseases (52–55).

Total LPC levels decreased throughout SIV infection (**Figure 2B**), compared to baseline levels, and remained decreased on ART, albeit their levels did not significantly change between the pretreatment and late ART levels (**Figure 2B**) thus suggesting that the SIV infection rather than ART is responsible for the changes in LPC levels.

**Figure 4:**
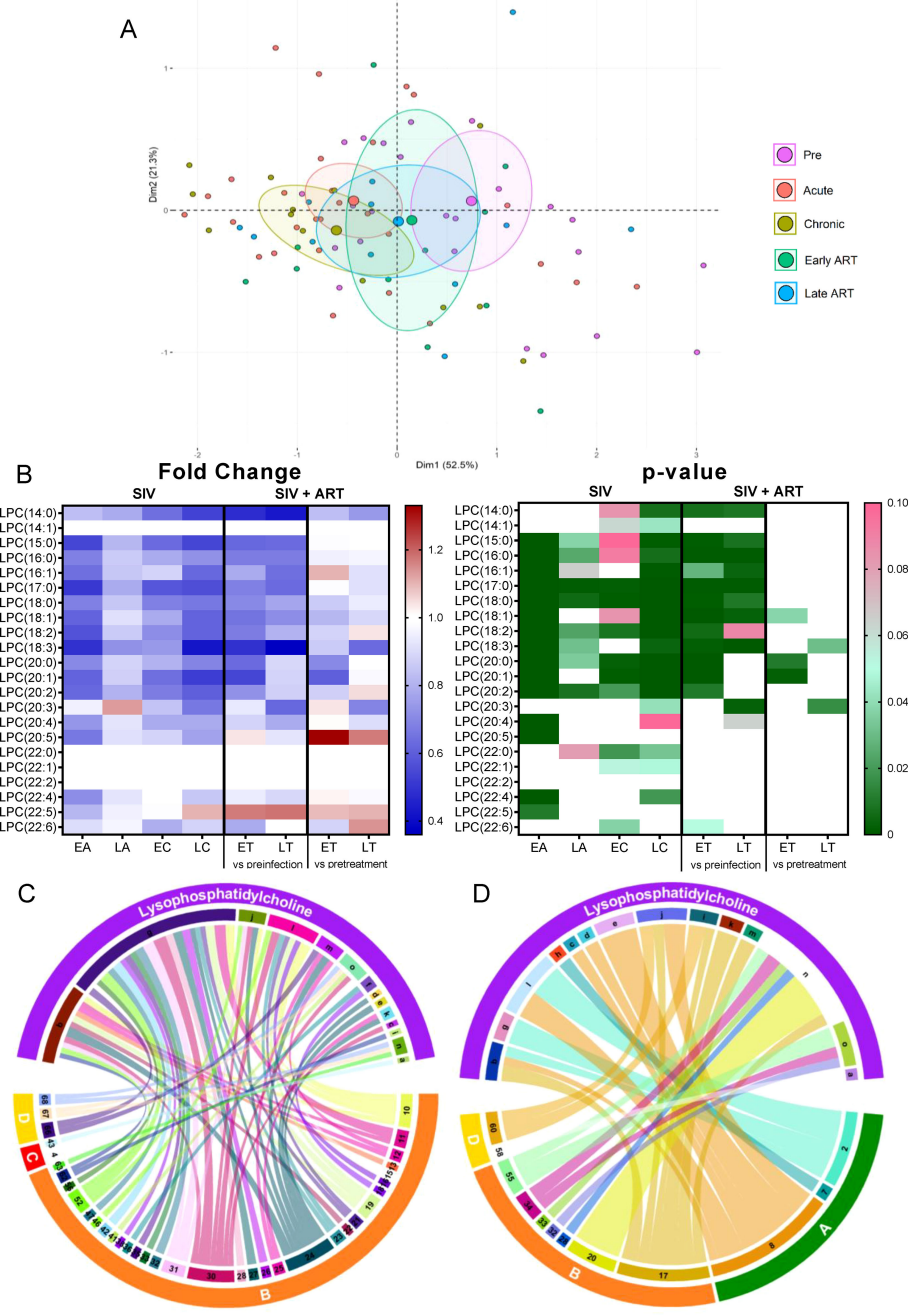
Principal component analysis (PCA) plot showing overall variation of lysophosphatidylcholines (LPC) based on concentrations of LPC species with confidence ellipses plotted for each group **(A)**. Groups are defined as preinfection, early acute infection (EA); late acute infection (LA), early chronic infection (EC), late chronic infection (LC), early ART (ET), late ART (LT). Heatmaps of fold changes and p-values of LPC species changes with SIV infection, where EA, LA, EC and LC are compared with preinfection, and changes with ART, where ET and LT are compared with preinfection, and then with pretreatment **(B)**. For fold change, red represents fold increase with deeper intensity indicating greater increase, and blue represents fold decrease with deeper intensity indicating greater decrease. White indicates no fold change. For p-value, green indicates statistically significant change (p<0.05) with deeper color intensity indicating stronger significance, and pink indicates trend to significance (p<0.1) with deeper color intensity indicating weaker significance. Positive **(C)** and negative **(D)** correlations between LPC species that are altered during SIV infection or with ART, represented in upper arc as: a: 14:0; b: 15:0; c: 16:0, d: 16:1; e: 17:0; f: 18:2; g: 18:3; h: 20:0; i: 20:1; j: 20:2; k: 20:4; l: 20:5; m: 22:4; n: 22:5; o: 22:6; and blood biomarkers of SIV disease progression and comorbidities: 2. CD4^+^ T cells (%);4. D-Dimer; 7. Neutrophils/μL; 8. Lymphocytes/μL; 10. Fibroblast growth factor (FGF); 11: IL-1B; 12. Granulocyte colony-stimulating factor (G-CSF); 13. IL-10; 15. Rantes; 16. IL-8; 17. IL-4; 18. CXCL9 [Monokine induced by gamma interferon (MIG)]; 19. CXCL10 (IP-10); 20. IL-2; 21. TNF-α; 22. IL-1RA; 23. Macrophage migration inhibitory factor (MIF); 24. I-TAC; 25. CCL22 [Macrophage-derived chemokine (MDC)]; 26. INF-γ; 27. Vascular endothelial growth factor (VEGF); 28. hepatocyte growth factor (HGF); 30. epidermal growth factor (EGF); 31. IL-15; 32. CCL-2 (monocyte chemoattractant protein-1, MCP-1); 33. CCL4 [macrophage inflammatory protein 1β (MIP-1β)]; 34. Granulocyte-macrophage colony-stimulating factor (GM-CSF); 35. CCL3 [macrophage inflammatory protein 1α (MIP-1α)]; 36. IL-17; 37. CCL11 (Eotaxin); 38. IL-6, 41. Lipopolysaccharide (LPS); 42. Soluble CD14 (sCD14); 43. Soluble Intracellular adhesion molecule-1 (1sICAM-1); 46. Neopterin; 47. sCD163; 52. CD38^+^ HLA-DR^+^ CD4^+^ T cells (%); 55. Ki-67^+^ CD8^+^ T cells (%); 56. CD38^+^ HLA-DR^+^ CD8^+^ T cells (%);58. Triglycerides; 60. Apolipoprotein A1 (apoA1); 66. Oxidized HDL (oxHDL), 67: Leptin; 68: Oxidized HDL (oxLDL). The biomarkers of SIV disease progression and comorbidities are represented on lower arc, and are grouped as: A: cell counts; B: T-cell immune activation/inflammation markers; C: coagulation markers; and D: atherogenic markers. Chords are plotted as a function of log of inverse of p-value (Anova). Greater the thickness of the chord, stronger the correlation.

On principal component analysis, LPC levels progressively clustered away from preinfection during acute and chronic SIV infection, these clustering shifts being lowered, but only partially reversed with ART (**Figure 4A**).

At individual level, 11/22 LPC species decreased throughout SIV infection (**Figure 4B**). ART successfully improved levels of only 3 of these 11 LPC species, the remaining ones showing persistent decreases on ART. The fact that the PTMs on ART showed slight increases of LPC species compared to the untreated group suggests that ART prevents further decreases of LPC in SIV-infected PTMs, however the treatment cannot normalize this lipid species to preinfection levels.

The LPC species that are decreased in SIV-infected PTMs [including LPC(15:0), LPC(16:0), LPC(16:1), LPC(17:0) etc], directly correlated with the levels of circulating CD4^+^ T cells and the lymphocyte counts (**Figure 4C**) and negatively correlated [including LPC(15:0), LPC(18:2), LPC(18:3), LPC(20:2) etc] with many inflammation/T-cell activation markers (**Figure 4D**), suggesting that their decrease may play a role in the increased inflammation and T-cell activation observed in SIV infection.

#### 2.3.3 Phosphatidylethanolamines

*Phosphatidylethanolamines (PEs)* are the second most abundant phospholipid in mammalian membranes (49), and a major component of the mitochondrial membrane, which can modulate mitochondrial functions such as production of energy (49). Total PE levels did not change with SIV infection, yet they significantly decreased with ART, as compared to both the preinfection baseline and pretreatment levels (**Figure 2B**).

**Figure 5:**
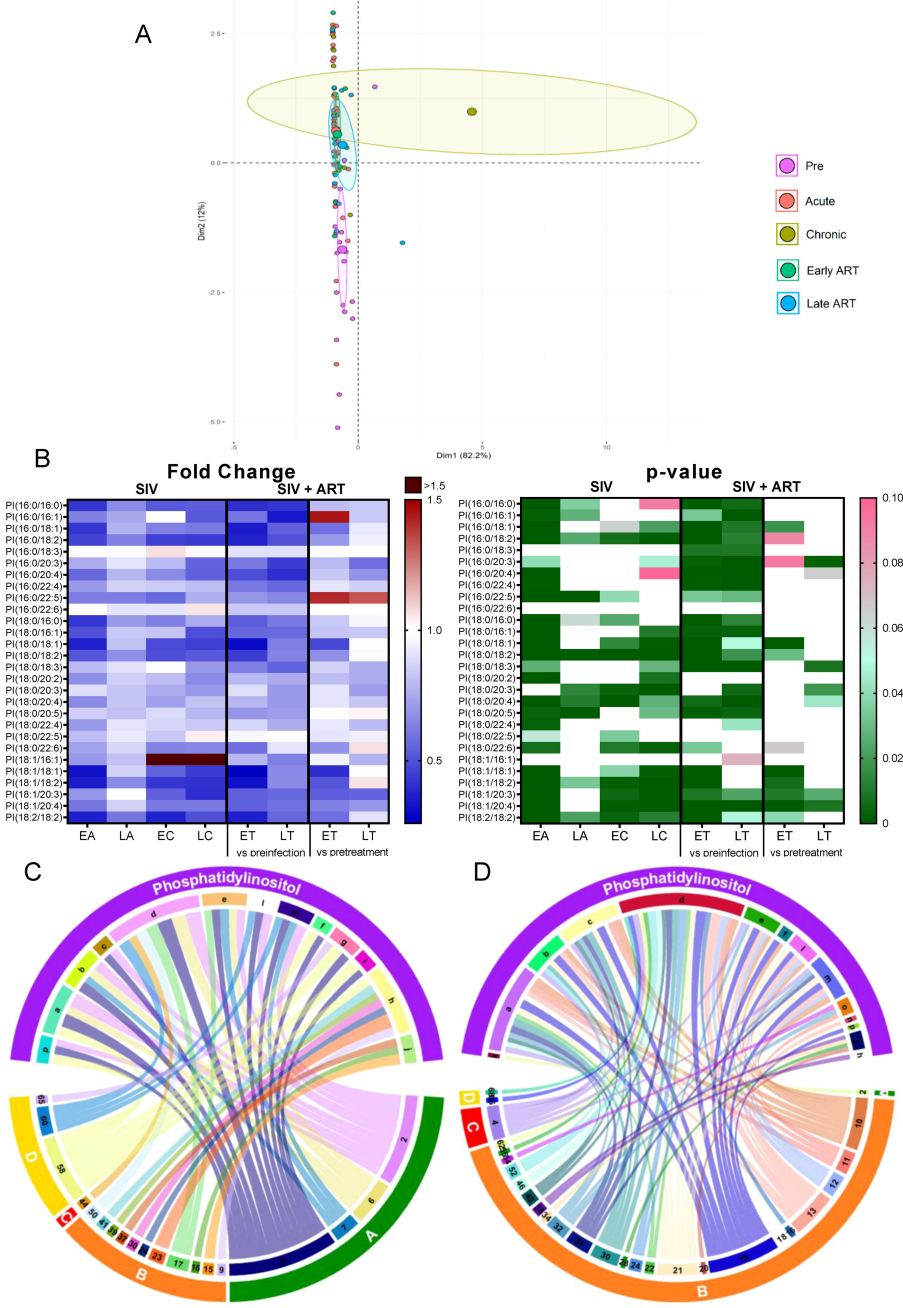
Principal component analysis (PCA) plot showing overall variation of phosphatidylethanolamines (PE) based on concentrations of PE species with confidence ellipses plotted for each group **(A)**. Groups are defined as preinfection, early acute infection (EA); late acute infection (LA), early chronic infection (EC), late chronic infection (LC), early ART (ET), late ART (LT). Heatmaps of fold changes and p-values of PE species changes with SIV infection, where EA, LA, EC and LC are compared with preinfection, and changes with ART, where ET and LT are compared with preinfection, and then with pretreatment **(B)**. For fold change, red represents fold increase with deeper intensity indicating greater increase, and blue represents fold decrease with deeper intensity indicating greater decrease. White indicates no fold change. For p-value, green indicates statistically significant change (p<0.05) with deeper color intensity indicating stronger significance, and pink indicates trend to significance (p<0.1) with deeper color intensity indicating weaker significance. Positive **(C)** and negative **(D)** correlations between PE species that are altered during SIV infection or with ART, represented in upper arc as: a: 16:0/16:0; b: 16:0/18:1; c: 16:0/18:2; d: 16:0/18:3; e: 16:0/20:1; f: 16:0/20:2; g: 16:0/20:3; h: 16:0/20:4; i: 16:0/22:4; j: 17:0/18:2; k: 17:0/20:4; l: 18:0/16:0; m: 18:0/18:1; n: 18:0/18:2; o: 18:0/18:3; p: 18:0/20:2; q: 18:0/20:3; r: 18:0/20:4; s: 18:1/16:1; t: 18:1/18:1; u: 18:1/18:2; v: 18:1/18:3; w: 18:1/20:3; x: 18:1/20:4; y: 18:2/18:2; z: Total species; and blood biomarkers of SIV disease progression and comorbidities: 2. CD4^+^ T cells (%);4. D-Dimer; 6. Platelets/μL; 8. Lymphocytes/μL; 10. Fibroblast growth factor (FGF); 11: IL-1B; 12. Granulocyte colony-stimulating factor (G-CSF); 13. IL-10; 15. Rantes; 16. IL-8; 17. IL-4; 18. CXCL9 [Monokine induced by gamma interferon (MIG)]; 19. CXCL10 (IP-10); 20. IL-2; 21. TNF-α; 22. IL-1RA; 23. Macrophage migration inhibitory factor (MIF); 24. I-TAC; 26. INF-γ; 27. Vascular endothelial growth factor (VEGF); 28. hepatocyte growth factor (HGF); 30. epidermal growth factor (EGF); 31. IL-15; 32. CCL-2 (monocyte chemoattractant protein-1, MCP-1); 33. CCL4 [macrophage inflammatory protein 1β (MIP-1β)]; 34. Granulocyte-macrophage colony-stimulating factor (GM-CSF); 35. CCL3 [macrophage inflammatory protein 1α (MIP-1α)]; 36. IL-17; 37. CCL11 (Eotaxin); 38. IL-6; 39. Soluble tissue factor (sTF); 40. p-selectin; 41. Lipopolysaccharide (LPS); 42. Soluble CD14 (sCD14); 46. Neopterin; 47. sCD163, 50. CD69^+^ CD4^+^ T cells (%); 51. Ki-67^+^ CD4^+^ T cells (%); 52. CD38^+^ HLA-DR^+^ CD4^+^ T cells (%); 53. CD25^+^ CD8^+^ T cells (%); 54. CD69^+^ CD8^+^ T cells (%); 57. Cholesterol; 58. Triglycerides; 59. low density lipoprotein (LDL); 60. Apolipoprotein A1 (apoA1); 61. Adiponectin; 65: high density lipoprotein (HDL); 66. Oxidized HDL (oxHDL); 67: Leptin; 68: Oxidized HDL (oxLDL). The biomarkers of SIV disease progression and comorbidities are represented on lower arc, and are grouped as: A: cell counts; B: T-cell immune activation/inflammation markers; C: coagulation markers; and D: atherogenic markers. Chords are plotted as a function of log of inverse of p-value (Anova). Greater the thickness of the chord, stronger the correlation.

On principal component analysis, progressive clustering away from preinfection occurred during acute and chronic SIV infection, and the shifting continued unabated on ART (**Figure 5A**).

The individual analysis of PE species showed that 13/71 species, unimpacted by SIV, significantly decreased with ART (**Figure 5B**). Twelve other PE species were inconsistently decreased (i.e., only on certain time points) with both SIV and ART. Their levels continued to decrease with ART, indicating that therapy boosts the virus effects. Therefore, we concluded that the variations seen in PE species were mainly due the effect of ART and not SIV.

Eleven PE species reduced on ART positively correlated with a several markers of inflammation/immune activation, such as CXCL-8 (IL-8), CCL-5 (RANTES), MIF, CCL-11 (eotaxin), and G-CSF, as well as with the T-cell counts and atherogenic biomarkers (**Figure 5C**). However, the majority of the PEs (≈30 species) which were reduced on ART, negatively correlated with numerous other inflammation/immune activation markers (**Figure 5D**). Therefore, we concluded that, while some PE species may exhibit inflammatory effects, many analyzed PE species, likely exhibit anti-inflammatory effects. Decrease of these anti-inflammatory PE species upon SIV infection may be another mechanism by which SIV causes increased inflammation and mitochondrial abnormalities.

We next performed detailed analyses of the major PE subclasses- PE-ether and PE-plasmalogen.

#### 2.3.4 PE-ethers

*PE-ethers (PE-O)* which are abundant in the mitochondria and endoplasmic reticulum (56), appeared slightly shifted from preinfection during the acute infection and early ART on the principal component analysis. Furthermore, they clustered away from each other during the chronic infection and late ART (**Supplementary Figure 1A**). The analysis of individual PE-O species revealed 3/27 to be inconsistently decreased with SIV infection and then with ART (as compared to pretreatment levels) (**Supplementary Figure 1B**), indicating that ART boosts the virus effects. One PE-O species [PE(O-16:0/20:4)] increased throughout SIV infection, but these increases were completely reversed with ART. Other PE-O species, [PE(O-18:0/18:1) and PE(O-18:0/18:2)] were drastically reduced by ART, pointing again to the strong impact of ART in reducing the PE levels and possibly inducing mitochondrial dysfunction.

PE-O (16:0/18:2), which was reduced on ART, positively correlated with oxLDL, leptin, HDL, triglycerides, MIF and CCL-5. PE-O(18:0/18:2) also reduced on ART, positively correlated with leptin, triglyceride, and CCL-5 (**Supplementary Figure 2A**). Both these PE-O species also negatively correlated with markers of inflammation and immune activation (**Supplementary Figure 2B**).

#### 2.3.5 PE-plasmalogens

*PE-plasmalogens (PE-P)* are glycerophospholipids containing a vinyl ether moiety and an esterified fatty acid, serving as inflammatory mediators with anti-oxidative properties (57). In our study, PE-Ps clustered on unique separate locations for preinfection, acute infection, chronic infection, early ART, and late ART on the principal component analysis (**Supplementary Figure 3A**).

Of the 50 individual PE-P species analyzed, 5 increased with SIV infection but were restored to preinfection levels on ART (**Supplementary Figure 3B**). Additionally, 3 species decreased under ART.

PE-P(16:0/20:3) and PE-P(18:1/20:3), increased with SIV, reversed with ART, and positively correlated with p-selectin and inflammation and immune activation markers (**Supplementary Figure 4A**). PE-P(16:0/20:3) and PE-P(18:1/20:3) negatively correlated with IL-4, GM-CSF; meanwhile, PE-P(18:1/20:3) negatively correlated with IL-6 and IL-17. IL-4 also negatively correlated with additional PE-P species (**Supplementary Figure 4B**).

#### 2.3.6 Lysophosphatidylethanoloamine

*Lysophosphatidylethanoloamine (LPE)* is a phosphatidylethanolamine metabolite (58), which increases intracellular calcium in normal and cancer cells (58–60). Intracellular calcium signaling plays an important role in the development from fertilization to organogenesis, and are also important regulators of neuronal functions ranging from early neuronal development to formation and maturation of neuronal circuits and long-term memory (61). In plants, LPE are reported to mitigate senescence progression (62).

Total LPEs decreased significantly only during early acute SIV infection, (**Figure 2B**), but this change was amplified by ART, with many post-ART samples yielding significantly lower levels than the pretreatment samples, which indicates that ART specifically impacts this class of lipids.

**Figure 6:**
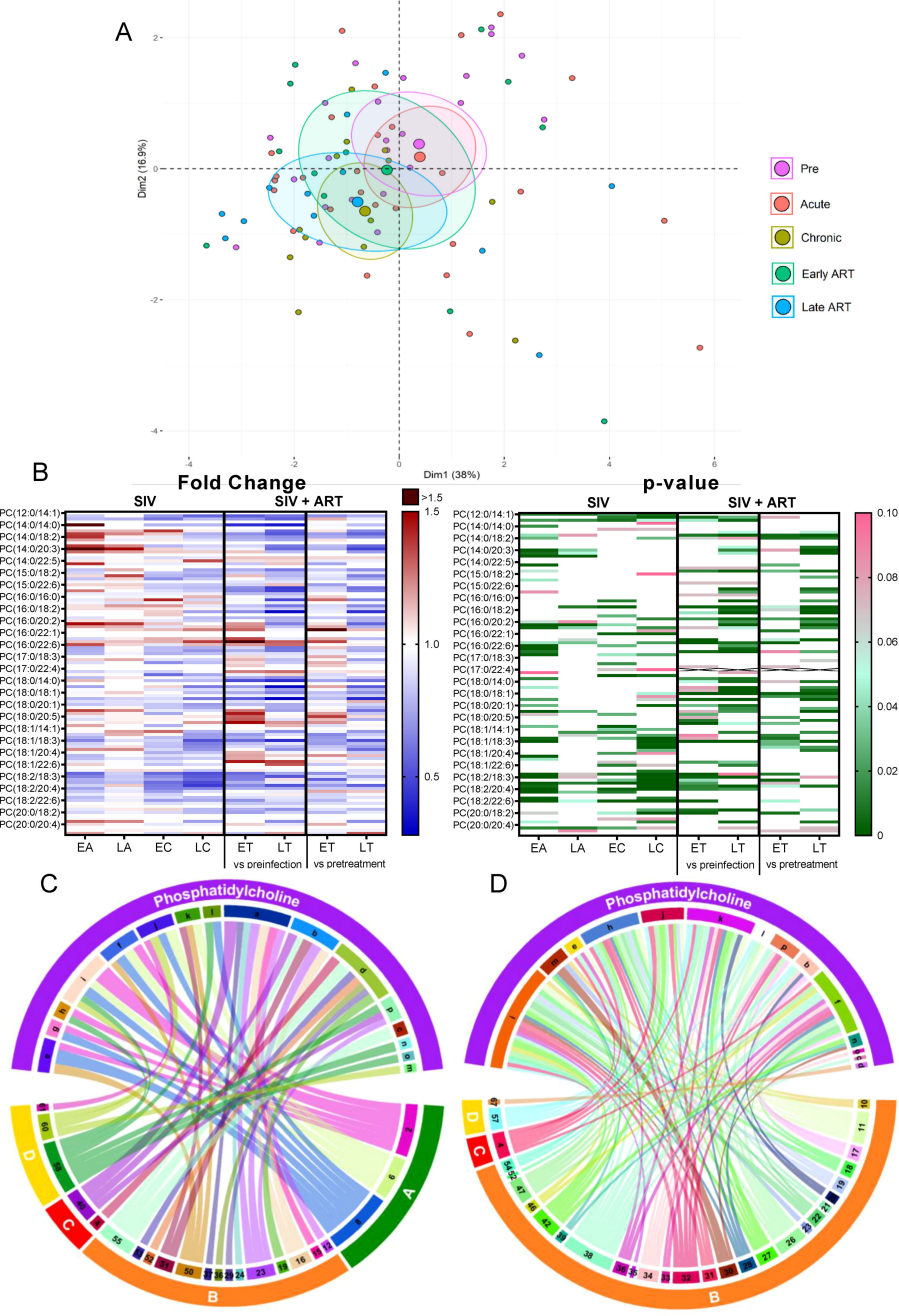
PCA plot showing overall variation of lysophosphatidylethanolamines (LPE) based on concentrations of LPE species with confidence ellipses plotted for each group **(A)**. Groups are defined as preinfection, early acute infection (EA); late acute infection (LA), early chronic infection (EC), late chronic infection (LC), early ART (ET), late ART (LT). Heatmaps of fold changes and p-values of lysophosphatidylethanolamine species changes with SIV infection, where EA, LA, EC and LC are compared with preinfection, and changes with ART, where ET and LT are compared with preinfection, and then with pretreatment **(B)**. For fold change, red represents fold increase with deeper intensity indicating greater increase, and blue represents fold decrease with deeper intensity indicating greater decrease. White indicates no fold change. For p-value, green indicates statistically significant change (p<0.05) with deeper color intensity indicating stronger significance, and pink indicates trend to significance (p<0.1) with deeper color intensity indicating weaker significance. Positive **(C)** and negative **(D)** correlations between LPE species that are altered during SIV infection or with ART, represented in upper arc as: a: 16:0; b: 16:1; c: 17:0; d: 18:0; e: 18:1; f: 18:2; g: 18:3; h: 20:0; i: 20:2; j: 20:3; k: 20:4; l: 22:6; m: Total species; and blood biomarkers of SIV disease progression and comorbidities coded as: 4. D-Dimer; 7. Neutrophils/μL; 8. Lymphocytes/μL; 11: IL-1B; 15. Rantes; 16. IL-8; 17. IL-4; 18. CXCL9 [Monokine induced by gamma interferon (MIG)]; 19. CXCL10 (IP-10); 20. IL-2; 22. IL-1RA; 23. Macrophage migration inhibitory factor (MIF); 24. I-TAC; 25. CCL22 [Macrophage-derived chemokine (MDC)]; 26. INF-γ; 27. Vascular endothelial growth factor (VEGF); 28. hepatocyte growth factor (HGF); 29. IL-5; 30. epidermal growth factor (EGF); 32. CCL-2 (monocyte chemoattractant protein-1, MCP-1); 34. Granulocyte-macrophage colony-stimulating factor (GM-CSF); 36. IL-17; 38. IL-6; 39. Soluble tissue factor (sTF); 40. p-selectin; 41. Lipopolysaccharide (LPS); 42. Soluble CD14 (sCD14); 47. sCD163; 50. CD69^+^ CD4^+^ T cells (%); 51. Ki-67^+^ CD4^+^ T cells (%); 52. CD38^+^ HLA-DR^+^ CD4^+^ T cells (%); 53. CD25^+^ CD8^+^ T cells (%); 54. CD69^+^ CD8^+^ T cells (%); 55. Ki-67^+^ CD8^+^ T cells (%);58. Triglycerides; 60. Apolipoprotein A1 (apoA1); 61. Adiponectin; 63. Alanine transaminase (ALT); 66. Oxidized HDL (oxHDL); 67: Leptin; 68: Oxidized HDL (oxLDL). The biomarkers of SIV disease progression and comorbidities are represented on lower arc, and are grouped as: A: cell counts; B: T-cell immune activation/inflammation markers; C: coagulation markers; and D: atherogenic markers. Chords are plotted as a function of log of inverse of p-value (Anova). Greater the thickness of the chord, stronger the correlation.

On principal component analysis, acute infection samples shifted from preinfection, yet late ART samples appeared to cluster in the vicinity of preinfection samples (**Figure 6A**).

Analysis of the individual LPE species found that 7/17 decreased during early acute SIV infection (**Figure 6B**), a trend which was generally reversed during late acute and early chronic infection. On ART however, all of them decreased again below the baseline levels. Four LPE species that did not change significantly with SIV decreased significantly from the pretreatment levels in PTMs on ART. Altogether, this indicates a strong impact of ART in decreasing LPE species.

Few LPE species increased with SIV and ART [i.e., LPE(22:5) and LPE(22:6)] and LPE(22:6) positively correlated with multiple inflammatory/immune activation markers and with ox LDL, thus suggesting that this species may be inflammatory and contribute to the SIV and ART induced inflammation. LPE(20:3), and LPE(20:4), which increased with SIV infection, positively correlated with p-selectin (**Figure 6C**), and thus may be causative factors in the increased thrombotic state observed in SIV/HIV.

Numerous other LPE species with lower carbon atoms, were however decreased with ART and negatively correlated with multiple inflammatory/immune activation markers, particularly with CXCL-9 (MIG). LPE(18:2) and LPE(17:0) correlated negatively with the greatest number of cytokines and chemokines (**Figure 6D**). These results indicate that LPE species may possess anti-inflammatory effects and that the decrease in these species during SIV and/or ART may contribute to increased inflammation. If the impact of these lipid species is indeed similar to the one reported in humans (63), their loss during SIV infection may also play a role in accelerated cellular aging.

#### 2.3.7 Phosphatidylinositols

*Phosphatidylinositols (PIs)* are minor components of the cell membrane, involved in signal transduction across the plasma membrane (64), through production of second messengers such as diacylglycerol, inositol1,4,5-triphosphate, phosphatidylinositol 3,4-bisphosphate and phosphatidylinositol 3,4,5-triphosphate (64).

Total PIs decreased throughout SIV infection and ART did not restore them to preinfection levels (**Figure 2B**); however, on ART, PI were maintained to pre-ART levels, suggesting that SIV infection has the strongest impact on this lipid class.

**Figure 7:**
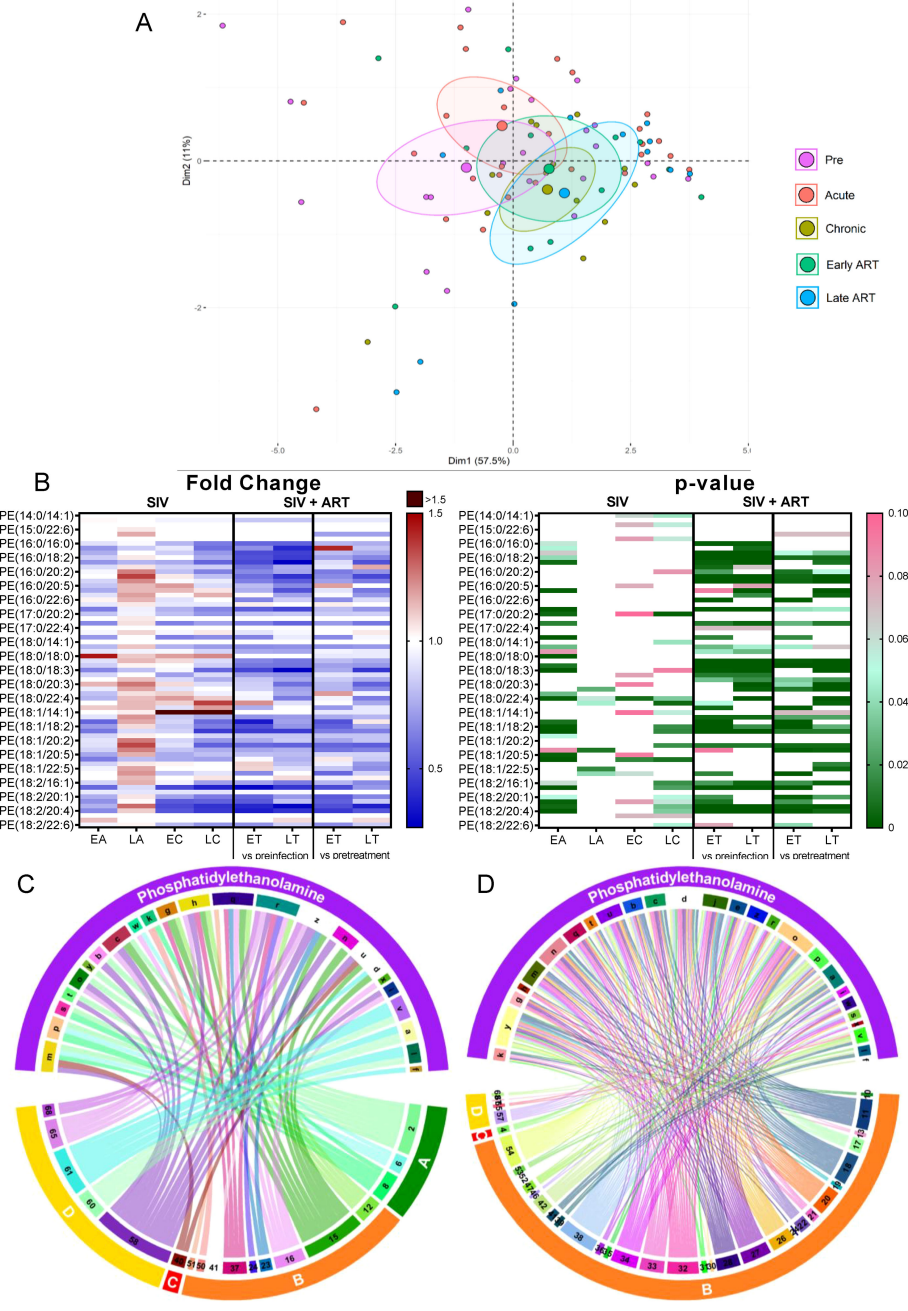
PCA plot showing overall variation of phosphatidylinositols (PI) based on concentrations of phosphatidylinositol species with confidence ellipses plotted for each group **(A)**. Groups are defined as preinfection, early acute infection (EA); late acute infection (LA), early chronic infection (EC), late chronic infection (LC), early ART (ET), late ART (LT). Heatmaps of fold changes and p-values of phosphatidylinositol species changes with SIV infection, where EA, LA, EC and LC are compared with preinfection, and changes with ART, where ET and LT are compared with preinfection, and then with pretreatment **(B)**. For fold change, red represents fold increase with deeper intensity indicating greater increase, and blue represents fold decrease with deeper intensity indicating greater decrease. White indicates no fold change. For p-value, green indicates statistically significant change (p<0.05) with deeper color intensity indicating stronger significance, and pink indicates trend to significance (p<0.1) with deeper color intensity indicating weaker significance. Positive **(C)** and negative **(D)** correlations between PI species that are altered during SIV infection or with ART, represented in upper arc as: a: 16:0/16:0; b: 16:0/18:1; c: 16:0/18:2; d: 18:0/16:0; e: 18:0/18:1; f: 18:0/18:2; g: 18:0/20:2; h: 18:0/20:3; i: 18:0/20:4; j: 18:0/20:5; k: 18:0/22:6; l: 18:1/18:1; m: 18:1/18:2; n: 18:1/20:4; o: 18:2/18:2; p: Total species; and blood biomarkers of SIV disease progression and comorbidities coded as: 2. CD4^+^ T cells (%); 4. D-Dimer; 6. Platelets/μL; 7. Neutrophils/μL; 8. Lymphocytes/μL; 9. C-reactive protein; 10. Fibroblast growth factor (FGF); 11. IL-1B; 12. Granulocyte colony-stimulating factor (G-CSF); 13. IL-10; 14. IL-12; 15. Rantes; 16. IL-8; 17. IL-4; 18. CXCL9 [Monokine induced by gamma interferon (MIG)]; 19. CXCL10 (IP-10); 20. IL-2; 21. TNF-α; 22. IL-1RA; 23. Macrophage migration inhibitory factor (MIF); 24. I-TAC; 28. hepatocyte growth factor (HGF); 30. epidermal growth factor (EGF); 31. IL-15; 32. CCL-2 (monocyte chemoattractant protein-1, MCP-1); 34. Granulocyte-macrophage colony-stimulating factor (GM-CSF); 37. CCL11 (Eotaxin); 38. IL-6; 39. Soluble tissue factor (sTF); 41. Lipopolysaccharide (LPS); 42. Soluble CD14 (sCD14); 46. Neopterin; 50. CD69^+^ CD4^+^ T cells (%); 52. CD38^+^ HLA-DR^+^ CD4^+^ T cells (%); 54. CD69^+^ CD8^+^ T cells (%); 55. Ki-67^+^ CD8^+^ T cells (%); 58. Triglycerides; 60. Apolipoprotein A1 (apoA1); 62. Aspartate transaminase (AST); 65: high density lipoprotein (HDL), 66. Oxidized HDL (oxHDL); 68: Oxidized HDL (oxLDL). The biomarkers of SIV disease progression and comorbidities are represented on lower arc, and are grouped as: A: cell counts; B: T-cell immune activation/inflammation markers; C: coagulation markers; and D: atherogenic markers. Chords are plotted as a function of log of inverse of p-value (Anova). Greater the thickness of the chord, stronger the correlation.

Principal component analysis identified the cluster shifting away from the preinfection group on both SIV-infected and ART samples (**Figure 7A**).

Among the individual PI species, 17/28 decreased throughout SIV infection (**Figure 7B**); ART reversed these trends in only 4/17 of these.

The PI species that decreased with SIV infection positively correlated with the lymphocyte, neutrophil, platelet, and CD4^+^ T cell counts (**Figure 7C**) and negatively correlated with IP-10 and FGF-basic. A fraction of these PIs also negatively correlated with inflammatory/immune activation markers and the coagulation marker D-dimer (**Figure 7D**). These results suggest that decreasing PI might contribute to the SIV-related inflammation/immune activation and hypercoagulation.

#### 2.3.8 Hexosylceramide

*Hexosylceramide (HCER)* is a bioactive glycosphingolipid synthesized from ceramide through addition of one or more sugar chains (65, 66). Sphingolipids and glycosphingolipids are reported to have signaling and regulatory functions and are involved in inflammation, angiogenesis and intracellular trafficking (67, 68).

Total HCER levels significantly increased throughout the SIV infection (**Figure 2B**). ART only partially normalized the HCER.

**Figure 8:**
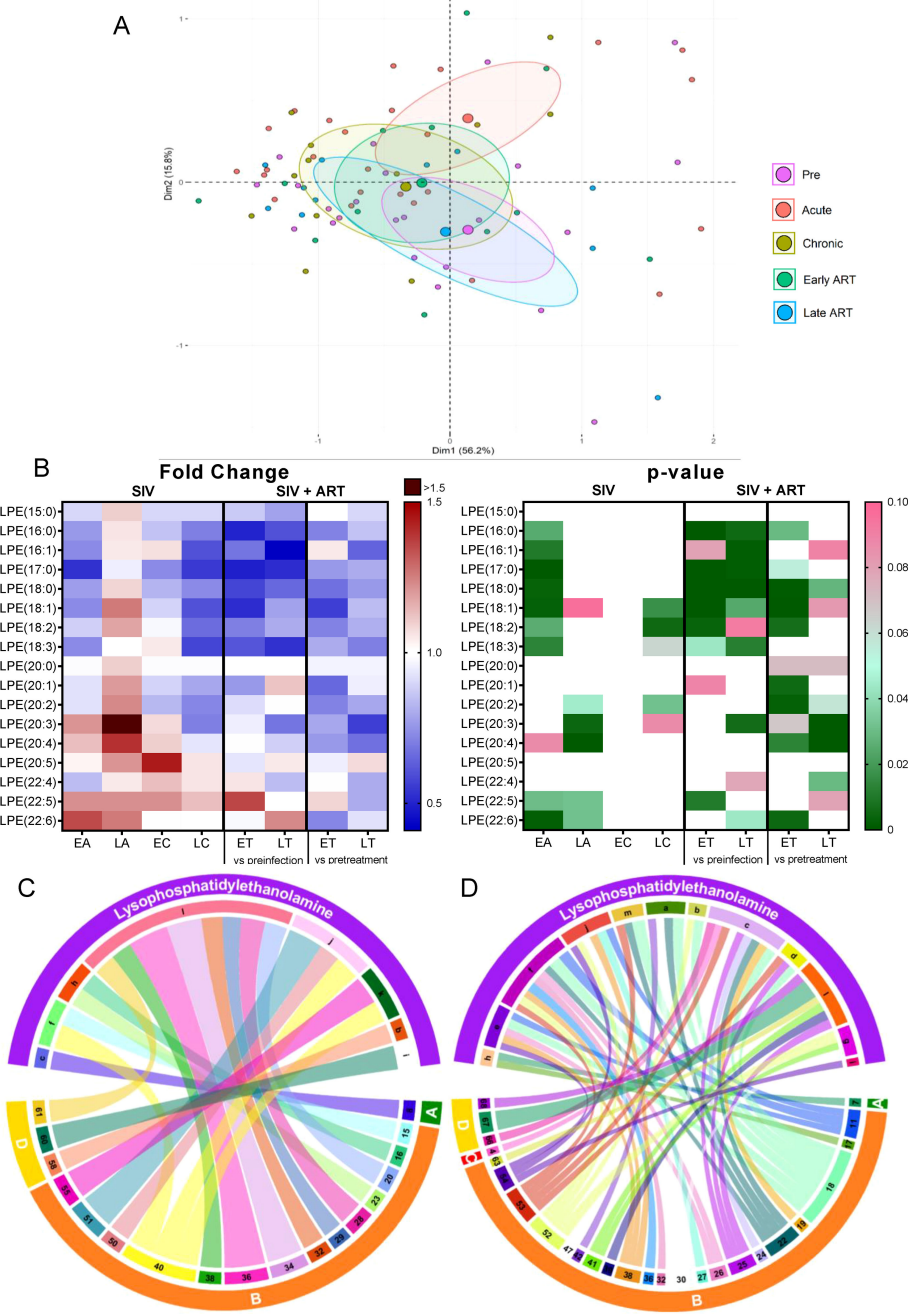
PCA plot showing overall variation of hexosylceramides (HCER) based on concentrations of hexosylceramide species with confidence ellipses plotted for each group **(A)**. Groups are defined as preinfection, early acute infection (EA); late acute infection (LA), early chronic infection (EC), late chronic infection (LC), early ART (ET), late ART (LT). Heatmaps of fold changes and p-values of hexosylceramide species changes with SIV infection, where EA, LA, EC and LC are compared with preinfection, and changes with ART, where ET and LT are compared with preinfection, and then with pretreatment **(B)**. For fold change, red represents fold increase with deeper intensity indicating greater increase, and blue represents fold decrease with deeper intensity indicating greater decrease. White indicates no fold change. For p-value, green indicates statistically significant change (p<0.05) with deeper color intensity indicating stronger significance, and pink indicates trend to significance (p<0.1) with deeper color intensity indicating weaker significance. Positive **(C)** and negative **(D)** correlations between HCER species that are altered during SIV infection or with ART, represented in upper arc as: a:14: 0; b: 16:0; c: 18:0; d: 22:0; e: 24:0; f: 24:1; g: Total species; and blood biomarkers of SIV disease progression and comorbidities coded as: 1. Viral loads; 5. Monocytes/μL; 15. Rantes; 16. IL-8; 17. IL-4; 20. IL-2; 21. TNF-α; 22. IL-1RA; 23. Macrophage migration inhibitory factor (MIF); 24. I-TAC; 38. IL-6, 39. Soluble tissue factor (sTF); 43. Soluble Intracellular adhesion molecule-1 (1sICAM-1); 44. Von Willebrand factor (vWF); 45. Platelet factor 4 (PF-4); 46. Neopterin; 47. sCD163; 53. CD25^+^ CD8^+^ T cells (%); 55. Ki-67^+^ CD8^+^ T cells (%); 60. Apolipoprotein A1 (apoA1); 61. Adiponectin; 62. Aspartate transaminase (AST); 68: Oxidized HDL (oxLDL). The biomarkers of SIV disease progression and comorbidities are represented on lower arc, and are grouped as: A: cell counts; B: T-cell immune activation/inflammation markers; C: coagulation markers; and D: atherogenic markers. Chords are plotted as a function of log of inverse of p-value (Anova). Greater the thickness of the chord, stronger the correlation.

Principal component analysis showed that, compared to the preinfection cluster, HCER species shifted the most during the acute infection, then progressively drifted back throughout the chronic infection, early ART, and late ART (**Figure 8A**).

The analyses of individual HCER species showed that 6/12 were significantly increased throughout SIV infection (**Figure 8B**), with only three being restored to preinfection levels by ART.

Six HCER species showing increases with SIV or with both SIV and ART positively correlated with markers of coagulation, adiponectin, and apoA1 lipoprotein (**Figure 8C**). HCER(24:1) and HCER(18:0), which were increased in SIV and ART, negatively correlated with markers of inflammation and immune activation (**Figure 8D**). We therefore concluded that, although this lipid class may be contributing to the hypercoagulation associated to SIV infection, some HCERs species may also reduce inflammation/immune activation and increase atheroprotective ApoA1 and adiponectin (lower levels of which contribute to insulin resistance in obesity (69)).

#### 2.3.9 Lactosylceramide

*Lactosylceramide (LCER)* is a glycosphingolipid formed by ceramide and a sugar moiety, which induces cellular responses such as production of CXCL-2 (MIP-2) and TNF (70, 71), activation of NF-kB (70), and oxidative burst and antimicrobial functions of leukocytes (72).

Total LCERs were elevated during SIV infection, particularly during the acute infection (**Figure 2B**); ART did not significantly reverse these increases.

On principal component analysis, farthest shifting from the preinfection cluster was observed on the late ART timepoint (**Supplementary Figure 5A**).

The analysis of individual LCER species showed that 6/12 increased significantly during the acute SIV infection (**Supplementary Figure 5B**); 1/12 LCER species increased throughout the infection and this trend was not reversed with ART.

LCER(16:0), which increased throughout SIV/ART, positively correlated with d-dimer and CD8^+^ T-cell immune activation markers (**Supplementary Figure 6A**), indicating that this species could be a contributing factor for the hypercoagulation and immune activation in SIV. LCER(24:1), which increased during acute SIV infection positively correlated with inflammation/immune activation markers, such as VEGF, HGF, CCL-4 (MIP-1B), IL-2, IL-1β and coagulation markers, such as von Willebrand factor (VWF) and sICAM-1 (**Supplementary Figure 6A**) and negatively correlated with other inflammation and immune activation markers, such as CCL-5, G-CSF, CXCL-8, I-TAC, CCL-1 (eotaxin) and also with HDL and oxLDL (**Supplementary Figure 6B**). This reflects on the complex interactions of LCER (24:1) with various inflammatory, coagulation, and lipid metabolism pathways.

### 2.4 Sphingomyelin

Sphingolipids are important signaling molecules, which are involved in cell growth and apoptosis (73). They are components of the cell membranes and their interaction with cholesterol is important for signal transduction (73). Sphingomyelin is a major membrane sphingolipid, that is a precursor of the bioactive ceramide and lysophospholipid (73) which modulates growth factor receptors and extracellular matrix proteins. Sphingolipids can be used as binding sites by microorganisms, viruses, and toxins (74).

Total SMs were significantly increased during acute SIV infection (**Figure 2B**) being reversed during chronic infection and ART.

On principal component analysis of SM species, acute infection shifted most significantly from the preinfection, with chronic infection cluster moving towards preinfection and the ART points forming distinct clusters (**Supplementary Figure 7A**).

Individual SM species were differently modified by both SIV and ART (**Supplementary Figure 7B**): 3/12 SM species increased throughout SIV infection and were minimally restored by ART; meanwhile, 2/12 species increased only during the acute SIV infection; one single SM species decreased with SIV infection, and was not significantly restored with ART; two SM species were not impacted by the SIV infection, but decreased on ART; finally, one species was not altered by the SIV infection, but significantly increased on ART.

SM (24:1), which increased with SIV infection and was normalized by prolonged ART, positively correlated with apoA1, coagulation markers like VWF and sICAM-1, and lymphocyte counts (**Supplementary Figure 8A**). SM(22:0), which decreased on ART when compared to pretreatment levels, negatively correlated with several markers of inflammation and immune activation (**Supplementary Figure 8B**). Thus, some SMs that are modified by SIV may promote hypercoagulation, while ART itself may impact other SMs that contribute to inflammation and immune activation.

## 3 Discussion

We assessed the lipidomic profiles in different stages of SIV infected of PTMs, and correlated them with disease progression and response to ART. Multiple *in vivo* and *in vitro* observations suggested that lipids are involved in driving HIV infection and replication (75). The correlations between specific lipid classes and species with inflammation and immune activation markers in various stages of HIV and ART support a contribution of lipidomic changes to HIV infection outcome (37–39, 76). Yet, establishing such correlations in PWH is confounded by multiple factors that impact lipid profiles: ART, diets and lifestyle. NHPs are in a “cleaner” metabolic state, receive uniformly healthy diets and housing, with virtually no behavioral confounding risk factors. Therefore, they are ideal for assessing the impact of HIV/SIV infection on lipidomics and, *vice versa*, the impact of the host lipid profiles on infection outcome. Further, NHP models may be used to assess the impact of ART and dietary interventions on plasma lipid profile.

We report that SIV infection and ART impacted specific lipid classes and species, pointing to potential pathways for SIV/ART-associated comorbidities. Many of these alterations were similar to those seen in PWH on ART: the overall decreases in serum LPCs (77, 78) and of specific lipid species (76, 77); increases in CE(22:4) (78); and elevations in serum levels of LCER and SM (79). Similarities with HIV infection support use of NHPs for testing the impact of ART and dietary interventions on lipid profiles. Meanwhile, we also identified differences between HIV and SIV with regard to their impact of on lipid profiles; thus, free fatty acids were only minimally altered in NHPs by both SIV infection and ART, while reported to be significantly impacted by HIV infection and ART in humans (76). Such interspecific differences are probably diet-related. Also, note that not all the different species measured within each lipid class exhibited changes in expression pattern. The significance of this observation is still to establish, as we still lack of complete understanding of lipid functions. Each lipid species is unique due to the combination of their headgroups and side chains, and thus different lipid species in the same class may exert different functions. This is also suggested by the observation that only certain lipid species within a given class correlate with pathologic processes (37, 39, 67, 80, 81). Also, different lipid species have different biological roles, likely as a result that different cell organelles have different lipid species composition (82). There are also several examples of biological processes that are dependent on few or even one lipid species. Alternatively, it is also possible that some of the closely related lipid species which are not altered by the HIV/SIV infections or ART could functionally replace the ones that are impacted by infection/ART. While current data are not sufficient to drive a definitive conclusion in depth analyses are warranted by these results.

In the absence of dietary and lifestyle confounding factors, we refined the lipidomic alterations and associated them with specific stages of infection and ART. These solid associations strongly suggest that lipid alterations were specific to either SIV infection or ART and could significantly drive and impact HIV/SIV-associated comorbidities. Note however, that ethical concerns requiring termination of studies once the endpoints are reached, limit the development of comorbidities in the NHP model. Therefore, in the following discussion we will refer to lipidomic changes that were observed in our study and were reported to be specifically associated with comorbidities in PWH or general population.

### 3.1 Cardiovascular diseases

Lipid changes could trigger CVD, which are specifically associated with progressive HIV/SIV infection (17). Here, SIV infection and/or ART induced changes in lipid species previously associated with atherosclerosis and CVD (83, 84), indicating that HIV-associated CVD could be linked to: (a) Increases in SMs, known to be associated with hypercholesterolemia, atherosclerotic lesions (85–88), and correlated with coronary disease severity (89, 90); (b) Decreases in total LPCs and specific LPCs species that are known biomarkers of coronary heart disease (91, 92), of incident myocardial infarction (92) and associated with increased CVD risk (81); (c) Increases of total LCERs and certain individual LCER species, known to induce hypertrophy in cardiomyocytes (93); (d) ART-induced reductions in PCs and CEs, known to relate to incident CVD (94, 95); (d) Changes by both SIV or ART of PE-plasmalogens, cholesteryl esters, dihydroceramides, hexosylceramides, lactosylceramides, and sphingomyelins which positively correlated with coagulation markers, pointing to possible involvement in the PWH’s and SIV-infected PTMs hypercoagulation. ART did not reverse many of these changes, and further induced deleterious lipid modifications, pointing to a persistent risk of CVD in HIV/SIV-infected individuals, irrespective of successful virus suppression with ART. As such, specific lipid changes induced by both the virus and ART could be responsible for CVD in PWH and for the high incidence of hypercoagulation and epicardial and myocardial inflammatory lesions observed in our model.

### 3.2 Diabetes

Other lipid changes that occurred in SIV-infected PTMs were reported in other models to be associated with insulin resistance and diabetes, two highly prevalent conditions in PWH (96, 97). These changes: (a) occurred with SIV infection, and persisted with ART: decreases in LPC species, which are highly predictive for impaired glucose tolerance and/or type 2 diabetes mellitus (98, 99); (b) occurred during acute SIV infection and further increased with ART: increases of DCER species that are emerging biomarkers of insulin resistance, altered glucose homeostasis (100), diabetes risk (101) and metabolic dysfunction (102); (c) increased throughout SIV infection and ART of the ceramide species associated with prediabetes (103) or higher insulinemia (103), and which are also independently and strongly predictive for CVD and mortality (104). All these are highly prevalent conditions in PWH (96, 97). Increases of plasma ceramides and their infiltration in tissues were reported to promote hypertension, atherosclerosis, fibrosis, apoptosis, diabetes, mitochondrial dysfunction and heart failure (104–107). Interventions to normalize ceramides in PWH might be useful to prevent diabetes, reduce the incidence of CVD, and possibly ameliorate other comorbidities. Finally, measuring plasma ceramide levels may represent a promising diagnostic tool for the PWH at risk for CVD and metabolic disturbances.

### 3.3 Weight alterations

Changes of lipid species seem to play a role in weight gain or loss: (a) Increases in PE species that positively correlates with obesity and decreases in the LPC and PC species that negatively correlate with the BMI were detected throughout infection and ART, suggesting that these lipid species may be involved in the weight gains seen in our PTMs on ART. Similar changes were reported in humans (108); (b) A reduction in PC(18:2/18:3), a subtype of PC(36:5), which was normalized by ART; and a decrease in long chain, polyunsaturated PCs during chronic SIV infection, many of which were not normalized by ART. This low PC signature may be due to reduced phosphatidylethanolamine methyltransferase (PEMT) activity, a metabolic alteration that may be responsible for the major weight loss observed in untreated PWH and SIV-infected PTMs. (c) Decreases in total and specific LPCs species which negatively correlate with the BMI in the PTMs gaining significant weight. These changes that persisted throughout SIV infection and ART were associated with obesity (109). (d) A profound and significant decrease of total and specific species of PI (that were reported to have anti-obesity effects (110)) particularly on ART, when most weight gain occurred in PTMs.

### 3.4 Inflammation

Our model is characterized by high levels of gut dysfunction and inflammation that persist under ART (47, 111). It is thus not surprising that lipid species with direct impact on inflammation were modified in SIV-infected PTMs: (a) A significant decrease in total PC levels occurred with prolonged ART. Several PC species, were also consistently decreased during chronic infection and after prolonged ART. Loss of PCs (known to have anti-inflammatory effects in intestinal cells (50)), with infection and ART suggests that this lipid class could play a role in the intestinal dysfunction and inflammation associated with HIV/SIV, and ART (112, 113). Therapeutic administration of PC could thus ameliorate gut health in PWH, as reported for patients with ulcerative colitis; (b) A profound and sustained decrease of both total LPCs and LPCs species occurred throughout SIV infection and ART. LPC levels decrease with inflammation and aging (114), their decrease being associated with poor prognostic and high mortality. LPCs reduce IL-6 production from macrophages post-LPS administration (115) and the platelet-activating factor (PAF) (116). LPCs can also inhibit neutrophil and eosinophil migration and activation and prevent tissue damage (117–119), enhance the suppressive platelet activity of Tregs (120, 121), increase extracellular antioxidant superoxide dismutase (122) and natriuretic peptide, which have anti-inflammatory and vasoprotective roles by binding C-reactive protein, thus decreasing its proatherogenic effects (123). LPCs also play a significant role in maintaining homeostatic T cell proliferation and support cytotoxic cell function postinfection (124). Their loss during SIV/HIV infection and ART could contribute to residual inflammation and immune dysfunction in PWH and SIV-infected PTMs. Our results suggest that LPC are excellent predictors of HIV/SIV disease progression, residual inflammation, immune dysfunction and comorbidities, particularly CVD. Efforts to revert the LPC losses via therapeutic interventions are thus warranted. (c) A significant decrease in the total levels of PC, PE and LPE was specifically induced by ART. These lipids have anti-inflammatory effects and inhibit macrophage-related inflammation (125–127). PC reduction in the intestinal mucosa was reported to occur in ulcerative colitis (49). Our data show that, in addition to the negative direct impact of SIV/HIV on the anti-inflammatory lipids, ART can also have a detrimental impact on lipids that are associated with gut integrity and systemic inflammation; (d) A significant decrease of phosphatidylinositol (PI), a pluripotent inhibitor of T-cell proliferation and activation and of inflammatory cytokines (Il-2, Th17) (128), throughout SIV infection and therapy. Dysregulation of PI signaling occurs in gastrointestinal malignancies and endoplasmic reticulum stress-mediated mucosal inflammation (129) and in both acute and chronic colitis (130). In PWH and SIV-infected PTMs on ART, PI dysregulation may be thus associated with the gut dysfunction and inflammation. PIs may therefore be potent biomarkers of residual gut dysfunction and inflammation in PWH. PI targeting is already a promising new, nontoxic nontherapeutic approach for inflammatory bowel disease (IBD) and their replenishment should be also targeted to improve clinical status and prevent comorbidities in PWH (131); (e) Increases in the ceramides (involved in multiple inflammatory processes, including obesity, diabetes, COPD, IBD and neuroinflammatory diseases (80, 132, 133)) by both SIV infection and ART. Altogether, our results illustrate the complex and vital role that lipids play in inflammatory pathways in HIV/SIV.

### 3.5 Mitochondrial dysfunction

Phospholipids, such as PC, PE and PI, which are the main components of the inner mitochondrial membrane (134), were significantly reduced in SIV-infected PTMs on ART. ART also reduced total and specific PE species, which maintain mitochondrial functions (49). As mitochondrial dysfunction plays a role in CVD/metabolic syndrome (135), diabetes (136) and neurodegenerative diseases (137), ART may be contributing to the CV, metabolic and neurodegenerative comorbidities seen in PWH and SIV-infected PTMs. The increases of LCER and other ceramides species which induce lipotoxicity in the pancreas and heart *via* mitochondrial damage (138–140) strongly support a role of this lipid class of lipids in promoting HIV/SIV-related comorbidities.

### 3.6 Neurocognitive abnormalities

Disturbances in the lipid levels induced by both virus infection and ART may contribute to the HIV/SIV-associated neurocognitive dysfunction. We report increases in the plasma levels of SM(18:0) with ART. SM(18:0) is a lipid species that was found to be increased in the cerebrospinal fluid of patients with Alzheimer’s disease-like pathology (141). Increases of TAG and MAG species occurred during early SIV infection. These lipids also increase in Alzheimer disease patients with mild cognitive impairment (142). Other neurologic abnormalities described in PWH can be triggered by changes in the lipid species described here: headache (143), which is associated with decreases in LPC species that also decreased in SIV-infected PTMs (144); depression, which associates PE, PC and PI decreases, and Cer, TAG and DAG increases, similar to those reported here (145, 146). It is thus conceivable that currently available or experimental therapies such as administration of high doses of Omega-3 fatty acids, of docosahehaenoic acid or of fermented soy bean lipid that can slow progression of Alzheimer disease, improves autisms, depression and schizophrenia or alleviate cognitive dysfunctions due to chemotherapy in human patients (147, 148) can be used as adjuvants to alleviate neurocognitive defects induced by infection and treatment in PWH.

In conclusion, SIV infection and ART have significant and distinctive impact on lipidome, which could contribute to non-AIDS comorbidities. By using a metabolically clean model, we identified specific lipidomic signatures of SIV infection or ART. We also described multiple lipid species alterations previously not reported by any of known diseases or clinical conditions, which may provide insight into the potential impact of lipid alterations in human diseases. Additional studies employing only antiretrovirals alone or in combinations could further help confirm the sole effects of ART on the lipidome, as previously shown with protease inhibitors (149). Our study strongly suggest that interventions aimed to supplement lipid deficits or block production of inflammatory lipids may help preventing comorbidities and may be employed as adjuvant treatments for HIV.

## 4 Materials and methods

### 4.1 Animals, infection and treatments

Archived samples were used from animals included in several previous studies conducted in the lab. Samples from twenty-five pigtailed macaques (*Macaca nemestrina*; PTMs) were used in this study. From ten PTMs we used only preinfection timepoints. Fifteen were intravenously infected with 300 tissue culture infectious doses (TCID50) of SIVsab92018. In eight of these PTMs SIV infection followed its natural course, while six PTMs received, starting at 1.5 mpi, the coformulated ART regimen with emtricitabine, tenofovir, and dolutegravir (48). ART was then administered throughout the follow-up (**Supplementary Table 1**).

All PTMs were housed and maintained at the Plum Borough Research animal facility of the University of Pittsburgh according to the standards of the Association for Assessment and Accreditation of Laboratory Animal Care (AAALAC). The experiments were approved by the University of Pittsburgh Institutional Animal Care and Use Committee (IACUC) (protocols 12040408, 15045829, 18021354, 20087378). The animals were fed and housed according to regulations set forth by the *Guide for the Care and Use of Laboratory Animals* and the Animal Welfare Act (150). The animals were paired and housed indoors in stainless steel cages. They had 12/12 light cycle, were fed twice daily, and water was provided ad libitum. Some of the environmental enrichment strategies that were employed include housing of animals in pairs, providing toys to manipulate, and playing entertainment videos in the animal rooms. The animals were observed twice daily, and any signs of disease or discomfort were reported to the veterinary staff for further evaluation. For sample collection, animals were anesthetized with 10 mg/kg ketamine HCl (Park-Davis, Morris Plains, NJ, USA) or 0.7mg/kg tiletamine HCl and zolazepan (Telazol, Fort Dodge Animal Health, Fort Dodge, IA) injected intramuscularly. At the completion of the study, the animals were sacrificed by intravenous administration of barbiturates.

### 4.2 Sample collection and processing

Blood was collected in EDTA anticoagulant collection tubes for use in this study. Preinfection and other critical time points after infection and treatment were selected, as shown in **Figure 1** and **Supplementary Table 1**: early acute infection (10 dpi), late acute infection ~1.5 months post infection (mpi), early chronic infection (~2.5mpi), and late chronic infection (~6mpi). For analysis of lipidome in SIV-infected PTMs on ART, they were sampled during early treatment (<6 months post-treatment; mpt) and late treatment (>6mpt). Plasma separated from all these samples by blood centrifugation for 20 minutes at 2,200 rpm was used for viral load measurements, lipidomic assays and assessment of inflammatory biomarkers. Whole blood samples were also used for the assessment of the complete blood counts (CBCs) by the Marshfield Laboratories.

### 4.3 Groups and analysis

To assess the lipidomic changes associated with the SIV infection and ART each timepoint was compared to the overall preinfection baseline. The samples collected from the PTMs on ART, were compared to both their preinfection baseline, as well to their pretreatment baseline (~1.5 months postinfection) to isolate effects primarily due to ART. In addition, comparisons were made between late and early ART to identify the effects of long-term ART. Furthermore, cross-sectional comparisons were done between treated and untreated groups at around ~6mpi.

### 4.4 Lipidomics platform

The data acquisition and analysis were performed by Metabolon. In brief, samples were prepared using the automated MicroLab STAR^®^ system from Hamilton Company. The method utilized a Waters ACQUITY ultra-performance liquid chromatography (UPLC) and a Thermo Scientific Q-Exactive high resolution/accurate mass spectrometer interfaced with a heated electrospray ionization (HESI-II) source and Orbitrap mass analyzer operated at 35,000 mass resolution. The MS analysis alternated between MS and data-dependent MS^n^ scans using dynamic exclusion. The hardware and software foundations for the informatics components were the LAN backbone, and a database server running Oracle 10.2.0.1 Enterprise Edition. Raw data was extracted, peak-identified and QC processed using Metabolon’s hardware and software. Peaks were quantified using area-under-the-curve. A data normalization step was performed to correct variation resulting from instrument inter-day tuning differences. Each compound was corrected in run-day blocks by registering the medians to equal one (1.00) and normalizing each data point proportionately. Lipids were extracted from the bio-fluid in the presence of deuterated internal standards using an automated BUME extraction according to the method of Lofgren et al. (151). The extracts were transferred to vials for infusion-MS analysis, performed on a Shimadzu LC with nano PEEK tubing and the Sciex SelexIon-5500 QTRAP. The samples were analyzed via both positive and negative mode electrospray. Individual lipid species were quantified by taking the ratio of the signal intensity of each target compound to that of its assigned internal standard, then multiplying by the concentration of internal standard added to the sample. Lipid class concentrations were calculated from the sum of all molecular species within a class, and fatty acid compositions were determined by calculating the proportion of each class comprised by individual fatty acids.

### 4.5 Nomenclature

The names of the individual lipid species are based on the length of the acyl chain(s) found in each lipid along with the number of unsaturated bonds found in each. Each acyl chain is denoted as (A:B) where: A= The total number of carbon atoms that are present in the chain. B= number of unsaturated bonds.

### 4.6 Flow cytometry

Whole blood was stained for flow cytometry, as described (152). Intracellular staining for Ki-67 was performed as described (153). Stained cells were acquired on an LSR-II flow cytometer (Becton Dickinson, BD, Franklin Lakes, NJ) and analyzed with FlowJo version 10 (Treestar, OR). Antibodies used were as follows (with clone in parenthesis). All antibodies are from BD unless otherwise noted: CD25-PE (2A3), CD8-PE-CF594 (RPA-T8), CD4 APC (L200), CD3 V450 (SP34-2), CD38 FITC (AT-1 Stemcell, Canada), CD69 APC-Cy7 (FN50), Ki-67 FITC (B56), HLA-DR PE-Cy7 (L243), CD45 PerCP (D058-1283). Trucount (BD) was used to quantify absolute CD4^+^ T cell counts, as per the manufacturer protocols.

### 4.7 Enzyme-linked immunosorbent assays

The following biomarkers were quantified with commercially-available kits as listed below: Leptin (MBS705354), Adiponectin (MBS069505), Omentin (MBS736583) all from MyBioSource (San Diego, CA). For sICAM-1 we used the kit BMS648INST from ThermoFisher (Waltham, MA); CRP was tested with the kit 2210-4 from Life Diagnostics (West Chester, PA); sCD163 was tested with the kit IQP-383 from Trillium Diagnostics (Brewer, ME); sTF was tested with the kit 845 from Sekisui Diagnostics (Burlington, MA); sCD14 was quantified with the kit DC140 from R&D (Minneapolis, MA), P-selectin was tested with the kit BMS650-4 from eBioscience (San Diego, CA), Apolipoprotein A1 and B were tested with the kits 3710-1HP-2 and 3715-1HP-2, both from Mabtech (Nacka Strand, Sweden); Oxidized HDL was tested with the kit STA-8880 from Cell Biolabs (San Diego, CA), Platelet Factor 4 was tested with the kit ELK-PF4 from RayBiotech (Peachtree Corners, GA); von Willebrand Factor and HDL & LDL/VLDL were tested with the kits ab223864 and ab65390, respectively, both from Abcam (Cambridge, United Kingdom). All these assays were run as per manufacturer’s protocols and our previous experience (42, 47, 154–158).

### 4.8 Cytokine testing

Was done on frozen plasma using a 29-plex luminex (Thermofisher, MA) as per manufacturer protocol and as previously described (155, 159, 160); results were read on a Bio-Plex reader (Bio-Rad Laboratories, CA).

### 4.9 Viral quantification

SIV pVLs were measured using quantitative real-time PCR, as described (152, 161, 162).

### 4.10 Statistical analyses

For the lipidomics data, standard statistical analyses were performed in ArrayStudio on log transformed data, by Metabolon. p<0.05 was considered statistically significant and p<0.1 was considered to indicate a trend toward significance. For lipidomic changes in SIV infection, statistical analysis between each timepoint and preinfection was done using ANOVA contrasts, and fold changes were calculated. For lipidomic changes on ART, statistical analysis between each timepoint and preinfection baseline, between each timepoint and pretreatment levels, and between late and early ART were done using ANOVA contrasts, and fold changes were calculated. For cross-sectional analysis between treated and untreated groups, statistical analyses were done using Welch’s Two-Sample t-Test. All heat maps were generated using Prism 8 (GraphPad, CA). All correlations were performed using Spearman’s ranked correlation in Prism 8 (GraphPad). All chord diagrams were generated using R v3.6 using the circlize package (163). All PCA calculations were performed using the prcomp function and plots were performed using the factoextra package of R.

## Data Availability

The raw data supporting the conclusions of this article will be made available by the authors, without undue reservation.
