# Supplementary material for: Plasma lipidomic alterations during pathogenic SIV infection with and without antiretroviral therapy
Source: Front Immunol. 2025 Mar 10;16:1475160. doi: 10.3389/fimmu.2025.1475160 (PMC11931036; doi:10.3389/fimmu.2025.1475160)
Supplement: Supplementary file 1 [file DataSheet1.pdf]

Plasma Lipidomic Alterations During Pathogenic SIV Infection with and Without Antiretroviral Therapy

Sindhuja Sivanandham,[1,2†¶] Ranjit Sivanandham,[1,2‡¶] Cuiling Xu,[1,2] Jen Symmonds,[1,6] Paola Sette,[1,2] Tianyu He,[1,2], Nick Funderburg[3], Mohamed Abdel Mohsen[4], Alan Landay[5], Cristian Apetrei,[2,6], & Ivona Pandrea[1,6*]

SUPPLEMENTAL TABLE AND FIGURES

Table. 1. Demographical, clinical and biological characteristics of the pigtailed macaques (PTMs) included in this study

PTM ID	SEX	AGE[1]	Δ WEIGHT[2]	Infection/treatment timepoints available (with CD4+ T cells and VLs[3])<br>BASELINE	10 DPI[4]	1.5 MPI[5]	2.5 MPI	>6 MPI
**SIV-infected, untreated**								
PTM177	M	4.2	NA[6]	1521 /µL				
PTM181	M	3.9	NA	2390 /µL				
PTM183	M	3.2	NA	2284 /µL				
PTM185	M	4.1	NA	1223 /µL				
PTM186	M	3.5	NA	2107 /µL				
PTM187	M	2.9	NA	2116 /µL				
PTM188	M	3.1	NA	2006 /µL				
PTM16	M	5.3	-0.6	1286 /µL			40 /µL<br>$6.93x10^{7}$/mL	
PTM20	M	4.2	1.7	1802 /µL			580 /µL<br>$1.49x10^{7}$ /mL	54 /µL<br>$9.28x10^{5}$ /mL
PTM21	M	4.1	1.3	1292 /µL			597 /µL<br>$6.04x10^{6}$/mL	65 /µL<br>$1.72x10^{6}$ /mL
PTM124	M	3.5	0.5	1273 /µL	989 /µL<br>$1.74x10^{8}$ /mL	207 /µL<br>$2.82x10^{7}$ /mL	58 /µL<br>$4.27x10^{7}$/mL	14 /µL<br>$3.41x10^{8}$ /mL
PTM125	M	3.9	0.2	1090 /µL	555 /µL<br>$7.84x10^{6}$ /mL	213 /µL<br>$2.69x10^{6}$ /mL	163 /µL<br>$2.73x10^{6}$ /mL	98 /µL<br>$7.12x10^{5}$ /mL
PTM126	M	3	0.2	1666 /µL	647 /µL<br>$5.81x10^{7}$/mL	32 /µL<br>$6.12x10^{8}$ /mL		
PTM127	M	3.6	0.5	3821 /µL	1262 /µL<br>$3.56x10^{8}$ /mL	626 /µL<br>$1.46x10^{6}$ /mL	582 /µL<br>$5.64x10^{5}$/mL	410 /µL<br>$6.88x10^{4}$/mL
PTM128	M	4	0.9	1606 /µL	505 /µL<br>$1.99x10^{8}$ /mL	75 /µL<br>$2.76x10^{6}$ /mL	94 /µL<br>$2.39x10^{6}$/mL	140 /µL<br>$1.31x10^{6}$ /mL
PTM174	M	5	0.6	1740 /µL	551 /µL<br>$9.4x10^{7}$ /mL			
PTM179	M	3	0.1	2045 /µL	467 /µL<br>$8.17x10^{7}$ /mL	124 /µL<br>$1.29x10^{8}$ /mL		
PTM180	M	2.7	-0.9	3272 /µL	1677 /µL<br>$1.94x10^{8}$ /mL	1051 /µL<br>$1.03x10^{7}$ /mL		
PTM59	M	4.5	-0.3	1716 /µL	711 /µL<br>$6.65x10^{6}$ /mL	37 /µL<br>$7.96x10^{7}$ /mL		
**SIV-infected, treated with antiretrovirals**								
				BASELINE	10 DPI	1.5 MPI	<6 MPT[7]	>6 MPT
PTM172	M	5.3	4.6	1715 /µL	359 /µL<br>$4.79x10^{7}$/mL	195 /µL<br>$5.89x10^{6}$ /mL	896 /µL<br><30/mL	626 /µL<br><30 /mL
PTM178	M	4.9	2.2	1301 /µL	396 /µL<br>$1.74x10^{7}$/mL	135 /µL<br>$1.6x10^{6}$ /mL	417 /µL<br>$1.75x10^{2}$/mL	373 /µL<br><30 /mL
PTM182	M	5.2	3.3	3224 /µL	1290 /µL<br>$7.52x10^{6}$ /mL	1184 /µL<br>$5.46x10^{4}$ /mL	1668 /µL<br><30 /mL	1267 /µL<br><30 /mL
PTM184	M	3.9	2.5	1324 /µL	676 /µL<br>$1.94x10^{7}$ /mL	585 /µL<br>$1.68x10^{6}$ /mL	524 /µL<br>$1.72x10^{2}$/mL	621 /µL<br><30 /mL
PTM42	M	6.9	3.4	1049 /µL	351 /µL<br>$1.34x10^{7}$ /mL	269 /µL<br>$3.39x10^{5}$ /mL	655 /µL<br><30	687 /µL<br><30 /mL
PTM60	M	4.5	3.2	1489 /µL	643 /µL<br>$2.82x10^{7}$/mL	317 /µL<br>$7.32x10^{6}$ /mL	645 /µL<br>$1.15x10^{5}$/mL	845 /µL<br><30 /mL

[1]Age at inclusion; [2]Δ WEIGHT is the weight variation between the inclusion and the completion; [3]CD4+ T cell counts are given per µL, while VLs are given in copies/mL; [4]DPI-day postinfection; [5]MPI-month postinfection; [6]NA-nonavailable (no follow-up); [7]MPT-months post-treatment

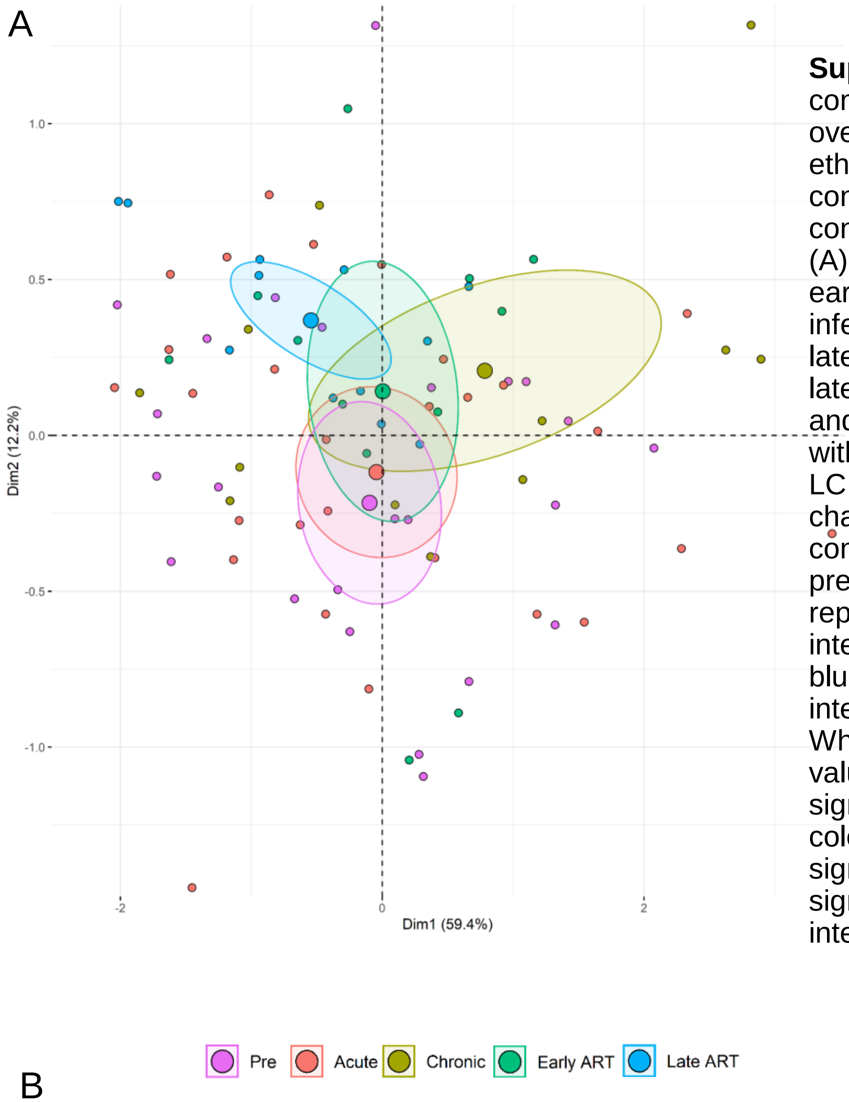

Supplemental Figure 1. Principal component analysis (PCA) plot showing overall variation of phosphatidyl-ethanolamine ethers (PE-O) based on concentrations of PE-O species with confidence ellipses plotted for each group (A). Groups are defined as preinfection, early acute infection (EA); late acute infection (LA), early chronic infection (EC), late chronic infection (LC), early ART (ET), late ART (LT). Heatmaps of fold changes and p-values in PE-O species changes with SIV infection, where EA, LA, EC and LC are compared with preinfection, and changes with ART, where ET and LT are compared with preinfection, and then with pretreatment (B). For fold change, red represents fold increase with deeper intensity indicating greater increase, and blue represents fold decrease with deeper intensity indicating greater decrease. White indicates no fold change. For p-value, green indicates statistically significant change (p<0.05) with deeper color intensity indicating stronger significance, and pink indicates trend to significance (p<0.1) with deeper color intensity indicating weaker significance.

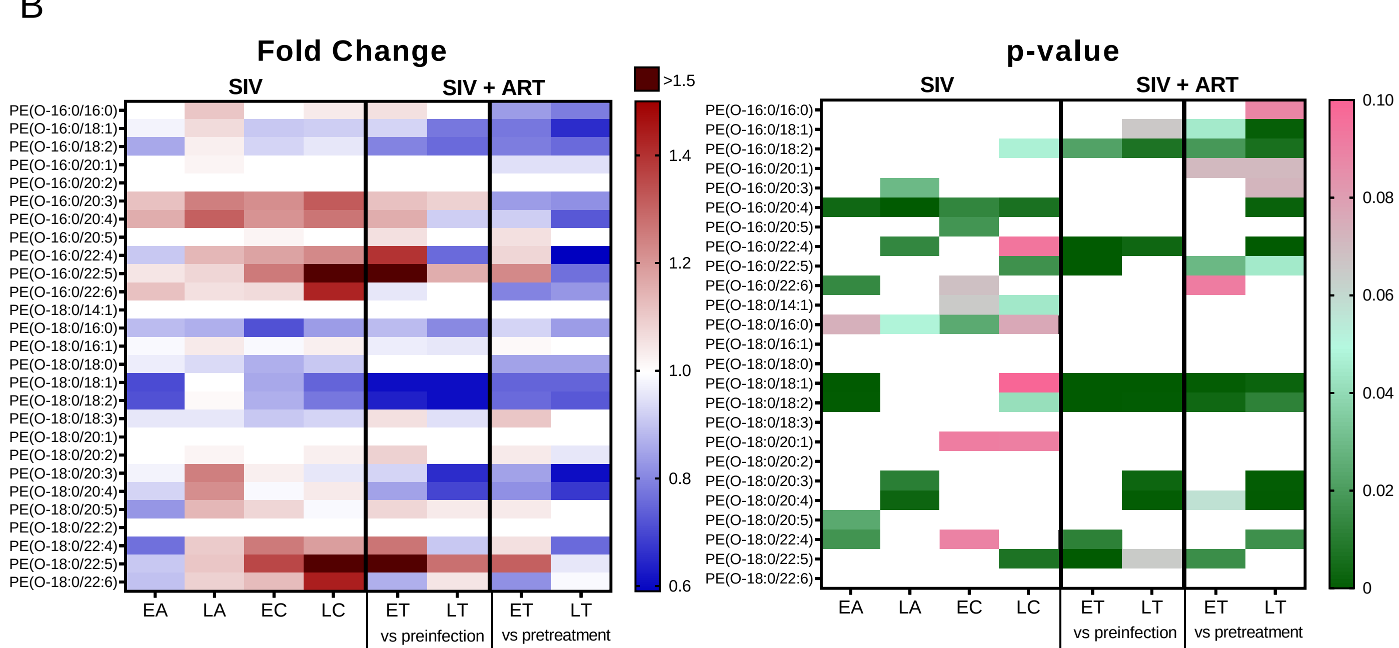

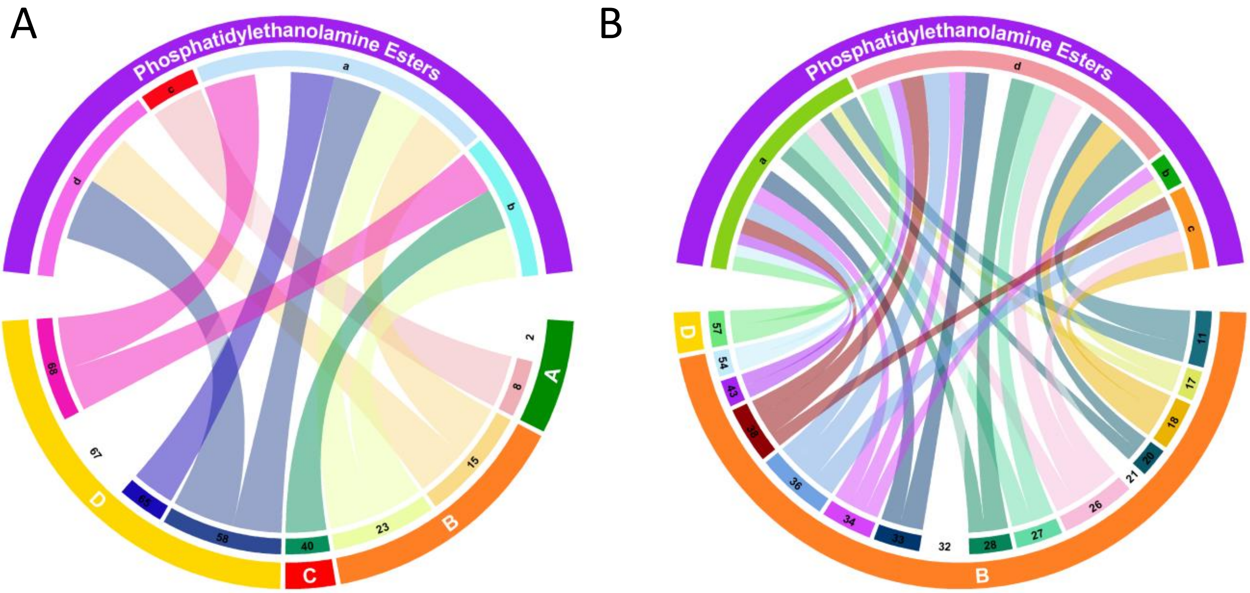

Supplemental figure 2. Positive (A) and negative (B) correlations between phosphatidylethanolamine esters (PE-O) species that are altered during SIV infection or with ART, represented in upper arc as: **a**: 16:0/18:2; b: 16:0/20:4; c: 18:0/18:1; d: 18:0/18:2; and blood biomarkers of SIV disease progression: 2. $CD4^+$ T cells (%); 8. Lymphocytes/μL; 11. IL-1B; 15. RANTES; 17. IL-4; 18. CXCL9 [Monokine induced by gamma interferon (MIG)]; 20. IL-2; 21. TNF-A; 23. Macrophage migration inhibitory factor (MIF); 26. INF-γ; 27. Vascular endothelial growth factor (VEGF); 28. Hepatocyte growth factor (HGF); 32. CCL2 (Monocyte chemoattractant protein-1, MCP-1); 33. CCL4 [Macrophage inflammatory protein 1β (MIP-1β)]; 34. Granulocyte-macrophage colony-stimulating factor (GM-CSF); 36. IL-17; 38. IL-6; 40. P-selectin; 43. Soluble Intracellular adhesion molecule-1 (sICAM-1); 54. $CD69^+$ $CD8^+$ T cells (%); 57. Cholesterol; 58. Triglycerides; 65. High density lipoprotein (HDL); 67. Leptin; 68. Oxidized LDL (oxLDL). The biomarkers of SIV disease progression are represented on the lower arc, and are grouped as: A: cell counts; B: T-cell immune activation/inflammation biomarkers; C: coagulation biomarkers; and D: atherogenic biomarkers. Chords are plotted as a function of log of inverse of p-value (Anova). Greater the thickness of the chord, stronger the correlation.

A

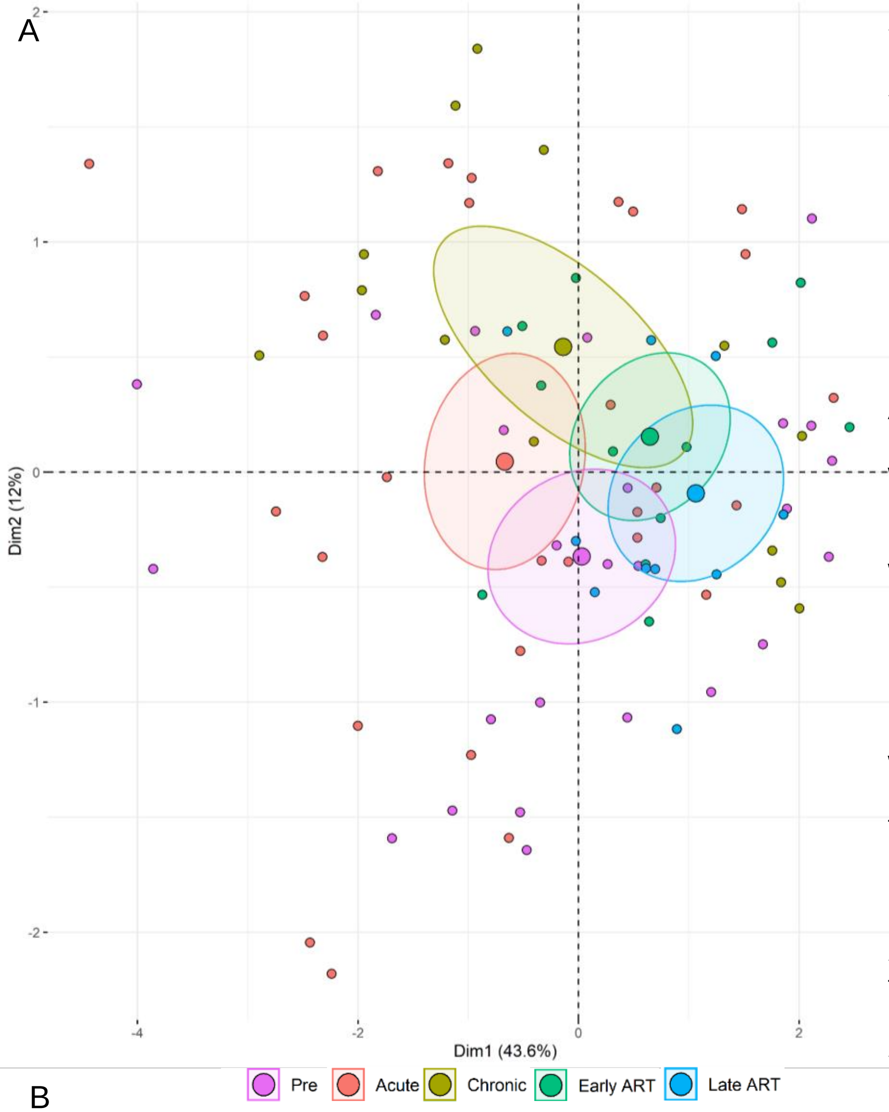

Supplemental Figure 3. Principal component analysis (PCA) plot showing overall variation of phosphatidylethanolamine plasmalogens (PE-P) based on concentrations of PE-P species with confidence ellipses plotted for each group (A). Groups are defined as preinfection, early acute infection (EA); late acute infection (LA), early chronic infection (EC), late chronic infection (LC), early ART (ET), late ART (LT). Heatmaps of fold changes and p-values PE-P species changes with SIV infection, where EA, LA, EC and LC are compared with preinfection, and changes with ART, where ET and LT are compared with preinfection, and then with pretreatment (B). For fold change, red represents fold increase with deeper intensity indicating greater increase, and blue represents fold decrease with deeper intensity indicating greater decrease. White indicates no fold change. For p-value, green indicates statistically significant change (p<0.05) with deeper color intensity indicating stronger significance, and pink indicates trend to significance (p<0.1) with deeper color intensity indicating weaker significance.

B

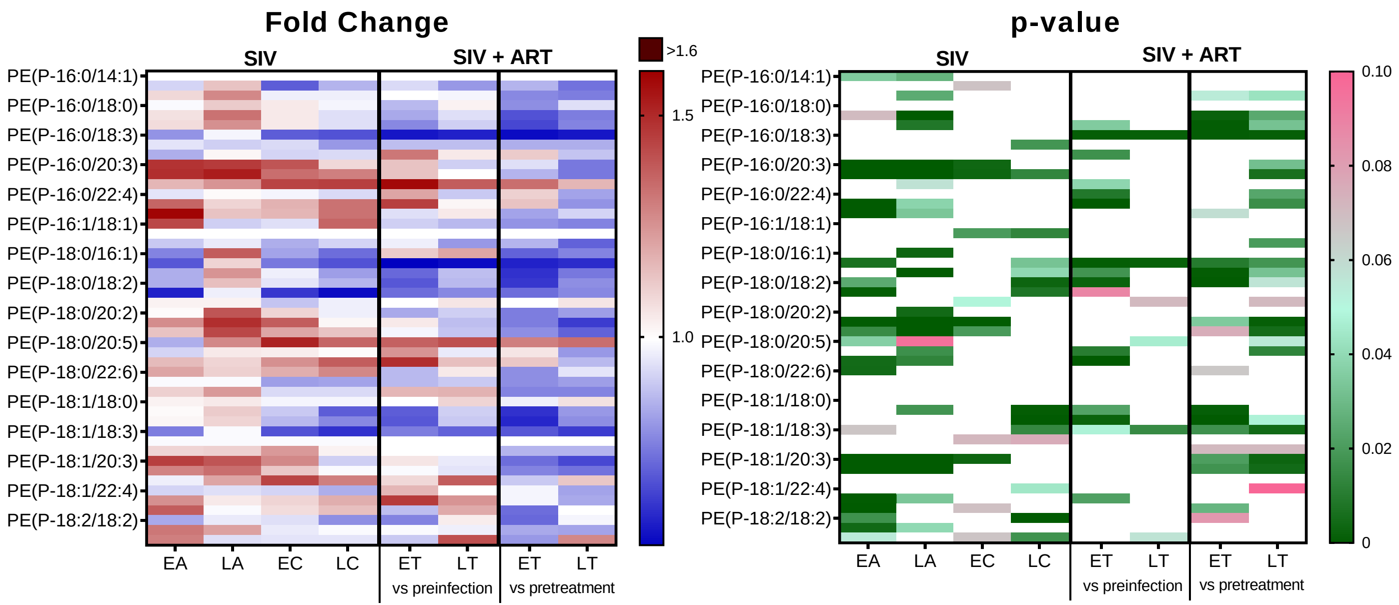

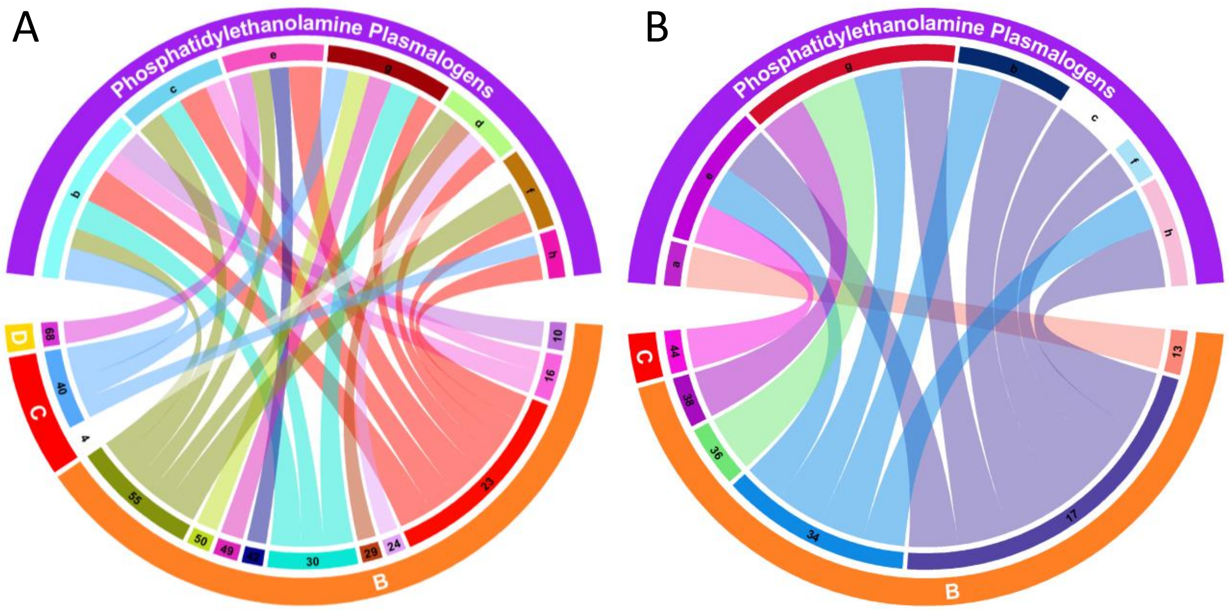

Supplemental Figure 4. Positive (A) and negative (B) correlations between phosphatidylethanolamine plasmalogens (PE-P) species that are altered during SIV infection or with ART, represented in upper arc as: a: 16:0/18:1; b: 16:0/20:3; c: 16:0/20:4; d: 16:0/22:5; e: 18:0/20:3; f: 18:0/20:4; g: 18:1/20:3; h: 18:2/20:4; and blood biomarkers of SIV disease progression coded as: 10. Fibroblast growth factor (FGF); 13. IL-10; 14. IL-12; 16. IL-8; 17. IL-4; 23. Macrophage migration inhibitory factor (MIF); 24. I-TAC; 29. IL-5; 30. Epidermal growth factor (EGF); 34. Granulocyte-macrophage colony-stimulating factor (GM-CSF); 36. IL-17; 38. IL-6; 42. Soluble CD14 (sCD14); 44. Von Willebrand factor (VWF); 49. $CD25^+$ $CD4^+$ T cells (%); 50. $CD69^+$ $CD4^+$ T cells (%); 55. Ki-67^+ $CD8^+$ T cells (%). The biomarkers of SIV disease progression are represented on lower arc, and are grouped as: A: cell counts; B: T-cell immune activation/inflammation biomarkers; C: coagulation biomarkers; and D: atherogenic biomarkers. Chords are plotted as a function of log of inverse of p-value (Anova). Greater the thickness of the chord, stronger the correlation.

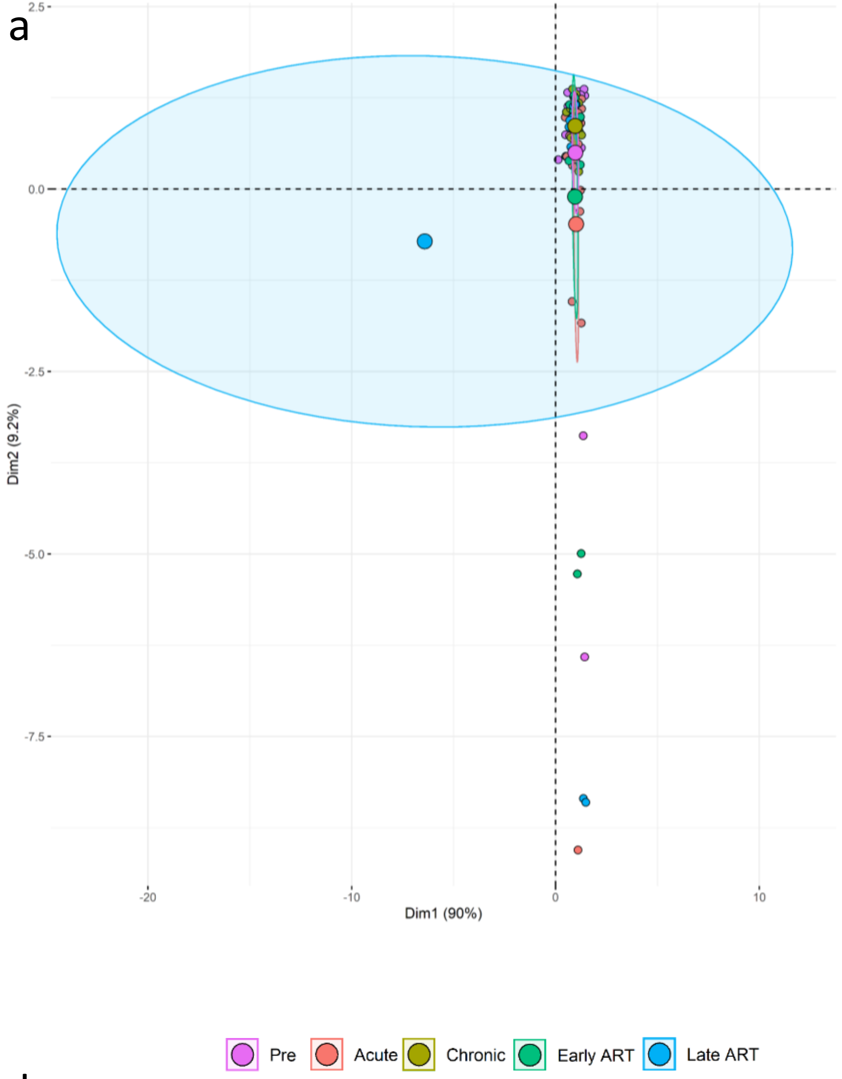

Supplemental Figure 5. Principal component analysis (PCA) plot showing overall variation of LCER based on concentrations of LCER species with confidence ellipses plotted for each group (A). Groups are defined as preinfection, early acute infection (EA); late acute infection (LA), early chronic infection (EC), late chronic infection (LC), early ART (ET), late ART (LT). Heatmaps of fold changes and p-values LCER species changes with SIV infection, where EA, LA, EC and LC are compared with preinfection, and changes with ART, where ET and LT are compared with preinfection, and then with pretreatment (B). For fold change, red represents fold increase with deeper intensity indicating greater increase, and blue represents fold decrease with deeper intensity indicating greater decrease. White indicates no fold change. For p-value, green indicates statistically significant change (p<0.05) with deeper color intensity indicating stronger significance, and pink indicates trend to significance (p<0.1) with deeper color intensity indicating weaker significance.

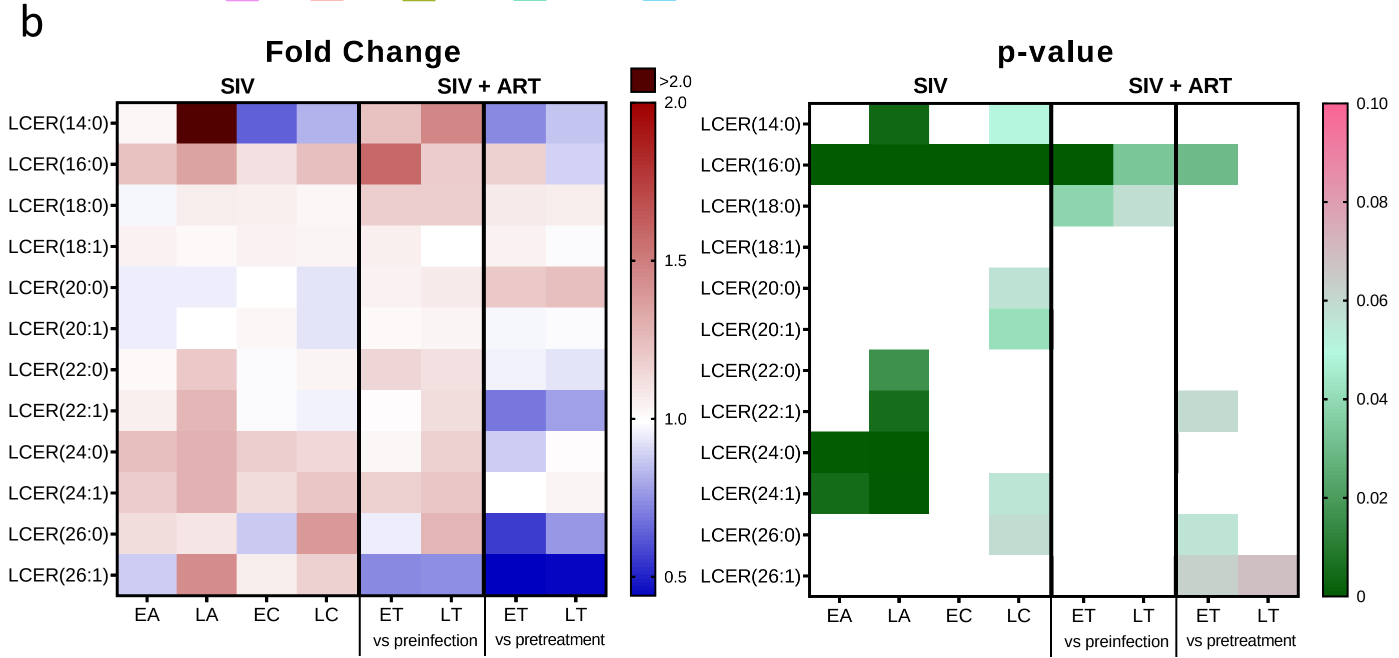

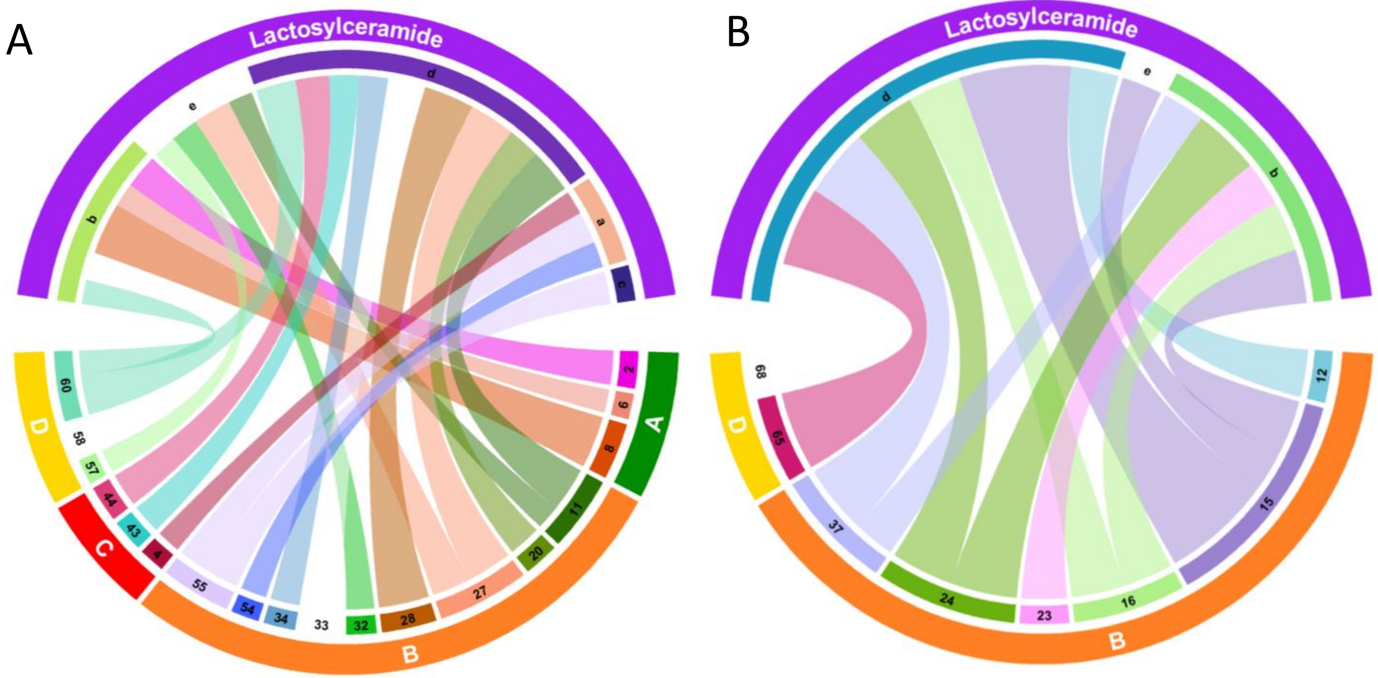

Supplemental Figure 6. Positive (A) and negative (B) correlations between lactosylceramide species that are altered during SIV infection or with ART, represented in upper arc as: a: 16:0; b: 18:0; c: 24:0; d:24:1; e: Total species; and blood biomarkers of SIV disease progression and comorbidities coded as: 2. CD4^{+} T cells (%); 6. Platelets/μL; 8. Lymphocytes/μL; 11. IL-1B; 12. Granulocyte colony-stimulating factor (G-CSF); 15. RANTES; 16. IL-8; 20. IL-2; 23. Macrophage migration inhibitory factor (MIF); 24. I-TAC; 27. Vascular endothelial growth factor (VEGF); 28. Hepatocyte growth factor (HGF); 32. CCL2 (Monocyte chemoattractant protein-1, MCP-1); 33. CCL4 [Macrophage inflammatory protein 1β (MIP-1β)]; 34. Granulocyte-macrophage colony-stimulating factor (GM-CSF); 37. CCL11 (Eotaxin-1); 54. CD69^{+} CD8^{+} T cells (%); 55. Ki-67^{+} CD8^{+} T cells (%); 65. High density lipoprotein (HDL); 68. Oxidized LDL (oxLDL). The biomarkers of SIV disease progression and comorbidities are represented on lower arc, and are grouped as: A: cell counts; B: T-cell immune activation/inflammation markers; C: coagulation markers; and D: atherogenic markers. Chords are plotted as a function of log of inverse of p-value (Anova). Greater the thickness of the chord, stronger the correlation.

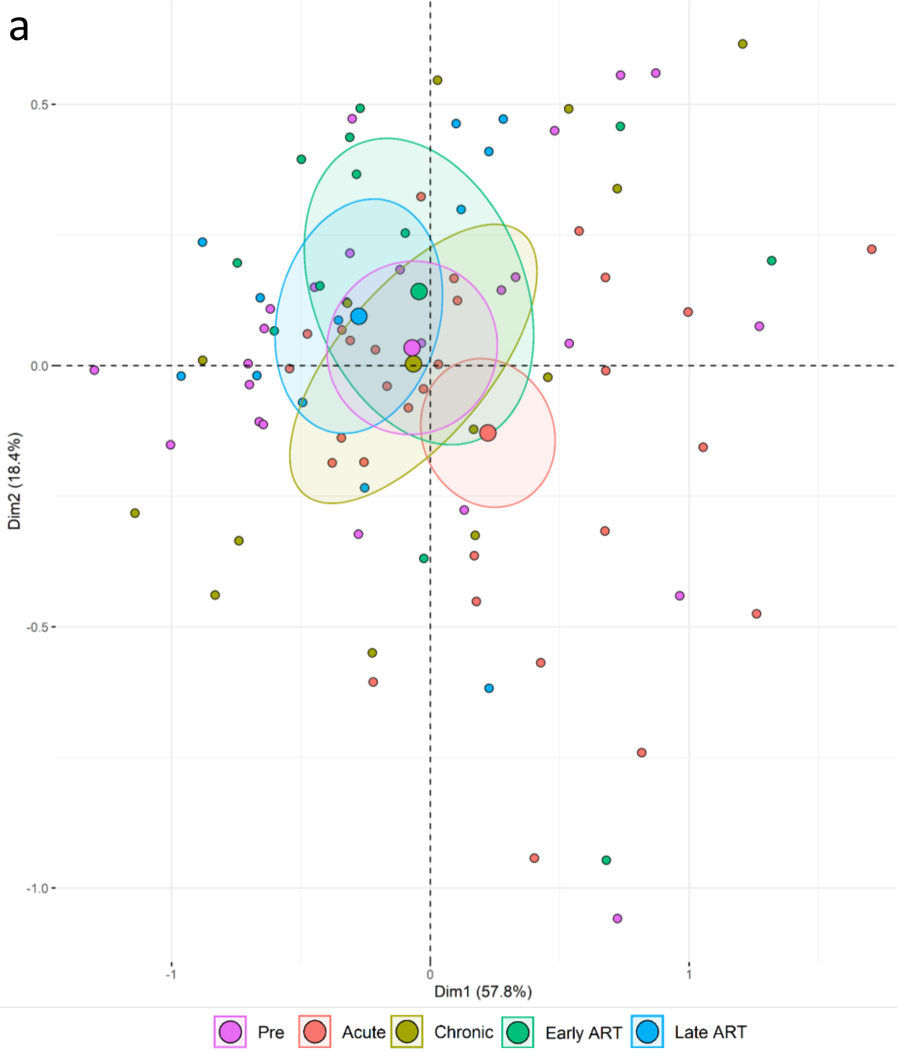

Supplemental Figure 7. PCA plot showing overall variation of sphingomyelins based on concentrations of sphingomyelin species with confidence ellipses plotted for each group (A). Groups are defined as preinfection, early acute infection (EA); late acute infection (LA), early chronic infection (EC), late chronic infection (LC), early ART (ET), late ART (LT). Heatmaps of fold changes and p-values for sphingomyelin species changes with SIV infection, where EA, LA, EC and LC are compared with preinfection, and changes with ART, where ET and LT are compared with preinfection, and then with pretreatment (B). For fold change, red represents fold increase with deeper intensity indicating greater increase, and blue represents fold decrease with deeper intensity indicating greater decrease. White indicates no fold change. For p-value, green indicates statistically significant change ($p<0.05$) with deeper color intensity indicating stronger significance, and pink indicates trend to significance ($p<0.1$) with deeper color intensity indicating weaker significance.

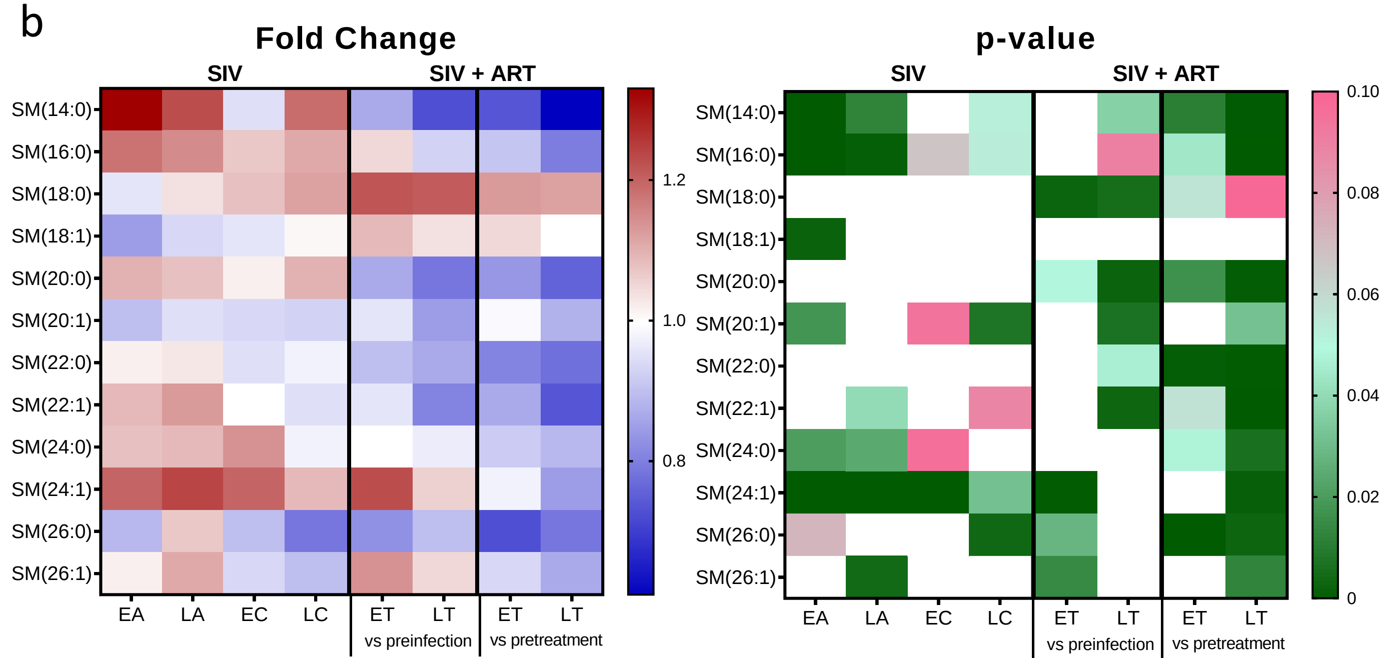

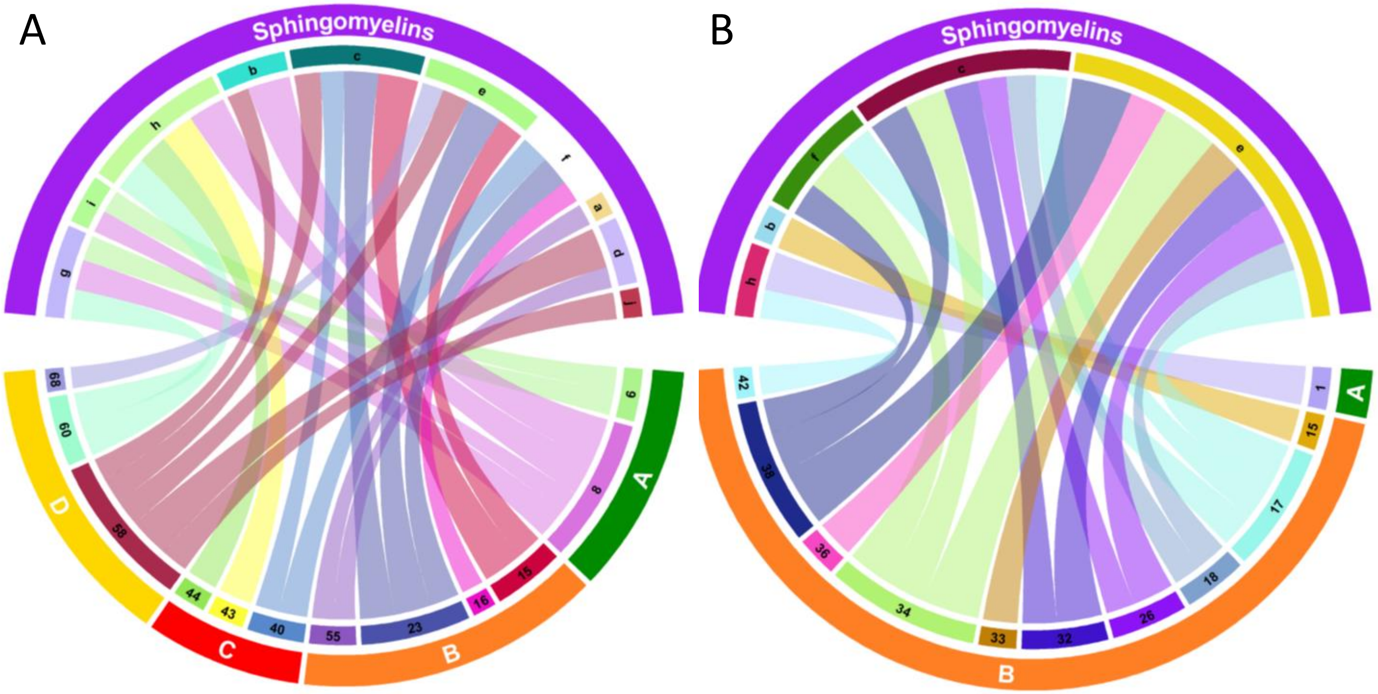

Supplemental Figure 8. Positive (A) and negative (B) correlations between sphingomyelin species that are altered during SIV infection or with ART, represented in upper arc as: a: 16:0; b: 18:0; c: 20:0; d: 20:1; e: 22:0; f: 22:1; g: 24:0; h: 24:1; i: 26:0; j: Total species; and blood biomarkers of SIV disease progression and comorbidities coded as: 1. Viral loads; 6. Platelets/μL; 8. Lymphocytes/μL; 15. RANTES; 16. IL-8; 17. IL-4; 18. CXCL9 [Monokine induced by gamma interferon (MIG)]; 23. Macrophage migration inhibitory factor (MIF); 26. INF-γ; 32. CCL2 [monocyte chemoattractant protein-1 (MCP-1)]; 33. CCL4 [macrophage inflammatory protein 1β (MIP-1β)]; 34. Granulocyte-macrophage colony-stimulating factor (GM-CSF); 36. IL-17; 38. IL-6; 40. p-selectin, 42. Soluble CD14 (sCD14); 43. Soluble Intracellular adhesion molecule-1 (1sICAM-1); 44. Von Willebrand factor (vWF); 55. Ki-67^{+} CD8^{+} T cells (%); 58. Triglycerides; 60. Apolipoprotein A1 (apoA1); 68: Oxidized HDL (oxLDL). The biomarkers of SIV disease progression and comorbidities are represented on lower arc, and are grouped as: A: cell counts; B: T-cell immune activation/ inflammation markers; C: coagulation markers; and D: atherogenic markers. Chords are plotted as a function of log of inverse of p-value (Anova). Greater the thickness of the chord, stronger the correlation.
